# You Say Potato, I Say Vegetable; You Say Tomato, I Say Fruit: Cognitive Validity of Food Group–Based Dietary Recall Questions^☆^

**DOI:** 10.1016/j.cdnut.2024.104502

**Published:** 2024-11-04

**Authors:** Anna W Herforth, Isabela F Sattamini, Deborah A Olarte, Pablo Diego-Rosell, Andrew Rzepa

**Affiliations:** 1 Department of Global Health and Population, Harvard T.H. Chan School of Public Health, Boston, MA, United States; 2 Division of Human Nutrition and Health, Wageningen University and Research, Wageningen, Netherlands; 3 Department of Nutrition, School of Public Health, University of São Paulo, Sao Paulo, Brazil; 4 Program in Nutrition, Department of Health Studies & Applied Educational Psychology, Teachers College, Columbia University, New York, NY, United States; 5 Department of Nutrition and Food Studies, New York University, New York, NY, United States; 6 Gallup, Washington, DC, United States; 7 Gallup, Inc., Dubai, United Arab Emirates

**Keywords:** diet quality questionnaire, DQQ, dietary diversity, minimum dietary diversity, MDD-W, dietary assessment, 24-h recall

## Abstract

**Background:**

There is a need for valid, standardized approach for list-based questionnaires to measure food group consumption for indicators of diet quality including dietary diversity.

**Objectives:**

A common method for collecting dietary diversity data consists of open-ended food group questions, e.g. “Yesterday, did you eat any vegetables, such as cucumber, cabbage, or celery?” We sought to examine the cognitive validity of open-ended questions that require respondents to categorize foods and closed-ended alternatives using sentinel foods.

**Methods:**

Pretesting and 83 cognitive interviews were conducted in 5 languages in São Paulo and New York City in 2018. In structured interviews, respondents were asked to describe their thought processes in answering each question. Their feedback and responses to closed-ended and open-ended food group questions were compared. The Gallup World Poll then piloted 2 versions of the questionnaire in a nationally representative sample of 1000 in Brazil in 2018.

**Results:**

Respondents in all settings miscategorized foods when asked open-ended food group questions (0%–82%, depending on the food group), respondents varied in their ability to think of other foods that belonged to specified food groups (35%–50% could think of any items), and open-ended questions presented an additional cognitive burden. There were no significant differences between the results from closed-ended and open-ended questions in the national pilot test. In the context of a multitopic survey, the finalized questionnaire took 3–5 min to answer, had no additional training requirements, and enumerators reported similar ease in administration as modules on other topics.

**Conclusions:**

For data collection on food group consumption, open-ended questions requiring respondents to categorize foods present cognitive validity problems. Closed-ended questions using sentinel foods reduce or eliminate ambiguity, presenting lower cognitive burden and greater comprehension. Based on these results, the closed-ended method has been adopted in international survey platforms for measuring dietary diversity and other aspects of diet quality.

## Introduction

For any new survey questionnaire module, cognitive testing is important to ensure data quality. Cognitive testing is standard practice in survey organizations such as Gallup, to ensure that respondents understand and can answer new questions. Cognitive testing consists of interviewing a small number of respondents (often <10) to understand and flag any problems with *1*) comprehension (does the respondent understand?), *2*) retrieval (does the respondent remember relevant information?), *3*) judgment (is the respondent able and willing to report true information?), and *d*) response (can the respondent provide an answer within the options given?) [1,2]. Questions that contain unknown terms, ambiguous concepts, or demanding recall or estimation abilities can affect cognitive validity of a survey module. Testing participants’ mental processes to answer the questions is a way to improve the quality of a survey by reducing response error that may occur if questions are misunderstood or misinterpreted [3].

In the context of survey research, cognitive validity refers to the extent to which the questions are generating the intended information [4]. It requires that respondents understand survey questions consistently: in the same way as each other and in the same way as the survey researcher intends. Furthermore, the ability to respond should not differ based on respondents’ pre-existing knowledge, education, or skills.

The Minimum Dietary Diversity for Women (MDD-W) was validated in 2014 as a proxy indicator of micronutrient adequacy for women aged 15–49 y and has since been used in many surveys, indicator frameworks, and programs [[5], [6], [7], [8], [9]]. The core attribute of this indicator (consumption of ≥5 of 10 predefined food groups) is that it could be calculated from food group consumption data rather than relying on complex, expensive quantitative dietary intake data. However, simplified methods for data collection had not undergone operational research and cognitive testing at the time the indicator was validated.

The MDD-W measurement guide offered 2 ways to collect the data: an open recall (listing all foods consumed in the previous 24 h, which would then later be classified into the correct food groups) or a list-based questionnaire. The model list-based questionnaire published in the 2016 guide included open-ended questions formulated as “Any [food group name], such as [example foods a, b, or c]?” (e.g., “Yesterday, did you eat any vegetables, such as cucumber, cabbage, or celery?”) [8]. Previous use of the questionnaire by the authors’ research group in Brazil, however, had indicated that may be problematic, because different respondents may have various ideas of what other foods belong in the category in question. Other research showed that misreporting using list-based open-ended questions was higher for some food groups than others [10,11].

It is well documented that people categorize foods differently. Food groups recognized in any particular culture depends on background, education, culture, and priorities. For example, the MDD-W food groups count potatoes as starchy staple foods. However, in some contexts (e.g., South Asia), many people consider potato to be a vegetable [12,13]. Indeed, it is botanically a vegetable, as a vegetative (non-reproductive) part of a plant. Tomato, on the contrary, is one of the most widely used vegetables in a culinary sense, but is botanically a fruit. A food anthropologist refers to the lack of a “vegetables” food group in Samoa: “tomatoes, cucumbers, or pumpkins, were not categorically recognized as a nutritional food group … they would ask for specific items like watercress or cucumbers, never referring to the category vegetable” [14](p436). In highland communities in Mexico and Guatemala, food categories in many people’s conception were related to harvest time, whether or not a food plant was wild, and whether it was used primarily for sustenance or for health; food categories were not based on nutrient content, culinary use, or botanical plant part [15]. Many cultural domain analysis studies, which ask participants to group foods and beverages that belong together, reveal disparate views of where items belong [16,17]. For example, one such study in Morocco [18] found consensus that dried fruits and nuts belong in the same group, in contrast to food groups used by nutritionists based on very different nutrient content of fruits and nuts; and fresh fruits and sweets were classified together as “dessert/sweet foods,” which was also observed in Uganda [19]. In Malawi, fresh oranges and orange-flavored soft drink were considered to be similar [20].

No single food classification system is correct. There are many reasonable ways people may categorize foods. Nutritionists designing food group–based questionnaires use particular food groups with the aim of correlating the foods consumed to nutrient intakes and health outcomes. Food groups based on nutrition science are useful for that purpose, but data collection must align with how respondents think. Asking open-ended questions and expecting respondents to consistently categorize food items into food groups in the same way as nutrition researchers may be wishful thinking. This concern is even stronger for global indicators, where researchers would depend on respondents across cultures, languages, and socioeconomic and education levels to categorize foods in the same way.

An alternative to open-ended list-based questions about food groups is to ask closed-ended questions comprising sentinel foods [21,22]. Sentinel foods are defined as the most frequently consumed items within a food group in a given population, which capture a large proportion of people who consumed the food group. They can be identified by analyzing dietary intake data, or if such data are unavailable, by interviewing key informants about the most commonly consumed items in a given setting [23]. For example, despite that many starchy roots and tubers are available in the United States, a single food item (potato) captures >95% of all people who consume that food group nationally. Therefore, a question that asked only the sentinel food “potato” would capture consumption of the “white starchy roots and tubers” food group, misclassifying <5% of people who consumed another item in the food group and not potato. Extending this principle, it was proposed that a short list of sentinel food items could capture each food group at a population level and may confer improved cognitive validity of questions and consistency in response.

The aim of this study was to test the cognitive validity of 2 forms of a food group-based questionnaire (open-ended, and closed-ended with sentinel foods) and to compare them and test their feasibility in a national multitopic survey setting. The desired outcome is a simple questionnaire to monitor diet quality, including the collection of data for MDD-W, which has cognitive validity and is low-burden for both respondents and enumerators.

## Methods

### Pretesting

To design the questionnaire, pretests were carried out among a convenience sample 9 respondents in the United States and Brazil; the pretest subjects were contacts of the authors who were not nutritionists or researchers. The aim was to narrow down the set of potentially unclear questionnaire questions to be included in cognitive testing. In the pretest, questions were formulated according to the model list-based questionnaire in the MDD-W measurement guide, which uses open-ended questions that ask respondents to report consumption of items that belong to the food group mentioned. The pretest highlighted food groups for which open-ended questions were clearly misunderstood or unnecessary and other food groups for which subjects’ understanding appeared to be more consistent. The latter were the focus of cognitive testing: dark green leafy vegetables (DGLVs), other vegetables, other fruits, and nuts and seeds, eggs, processed meats, deep-fried foods, salty packaged snacks, soft drinks, and fast food. The pretests also tested 2 forms of an introduction: short and long.

### Cognitive testing

Cognitive testing was carried out through 83 cognitive interviews in 5 languages (Portuguese, English, Chinese, Arabic, and Farsi) in São Paulo and New York City from April to May 2018. In these interviews, we compared responses to closed-ended sentinel food questions to open-ended food group questions described in the 2016 MDD-W measurement guide [8]. The interviews were designed to understand how questions about food group consumption in the previous day are understood by respondents and whether there were issues with comprehension, retrieval, judgment, or response.

Semistructured interviews were conducted using retrospective verbal probing techniques. Each interview consisted of 2 parts: *1*) the respondent answered the questionnaire and *2*) the interviewer probed the respondent to report on their interpretation of meaning of the questionnaire items and their answers to the items. Two different versions of the survey questionnaire were developed to ask about potentially ambiguous food groups, and to try “stem questions” before a set of several food group questions (e.g., “Yesterday, did you eat any of the following fruits: …”) . In the interviews, when a respondent answered yes to a food group question, specific follow up questions were asked about what the food consumed was and whether it was consumed as part of a mixed dish or by itself. For especially diverse food groups (fruits, vegetables, nuts, and seeds), respondents were also asked for their feedback on whether they preferred a shorter or longer list of examples (each read out) and why. Both versions included a series of retrospective questions about overall ease or potential ambiguity, demographic information. Informed consent was obtained before each interview. The versions tested are in the Supplemental Material.

Cognitive interviews were conducted by the University of São Paulo Nutrition Department, within the Faculty of Public Health, and by the Columbia University Teachers College Nutrition Education Department in New York City. The 6 interviewers in Brazil and 9 interviewers in New York were all postgraduate students in nutrition experienced in dietary topics and probing.

In Brazil, interviews were carried out in 2 municipal health centers in the city of São Paulo and at an agricultural cooperative workers association center in the rural district of Parelheiros. These centers cater to Brazilian residents hailing from a wide variety of locations, and the interviews included Brazilians from each region of Brazil except for the Amazonas state (Supplemental Material).

In New York City, respondents from 4 different nations were surveyed: the United States, China, Egypt, and Iran. Situating cognitive testing in New York City enabled interviews with recent immigrants who closely retained their language and eating habits from their country of origin. Based on the interviewers’ language abilities, nationalities were selected that could be represented or proxied by recent immigrants to New York City who live in communities dominated by their nationality of origin, and who could be interviewed in their native language by ≥1 student who is fluent or highly proficient in the language. For the interviews conducted in Chinese, Arabic, and Farsi, an acculturation scale was developed, adapted from the Suinn-Lew Asian Self-Identity Acculturation Scale [24], to screen respondents for minimal acculturation (Supplemental Material). The scale included 8 questions including identification of the language in which the respondents read and think, as well as the foods usually eaten at home and away from home. To participate in the interview, 2 qualifying questions were asked of the participants: whether the potential interviewee is fluent in the language of the questionnaire and if they “only” or “mostly” eat the food from their country of origin.

All interviews were conducted by teams of 2. One member who was the designated interviewer and another was the designated observer and recorder. Each interview team administered an equal number of the 2 versions of the questionnaires. All responses were recorded on paper questionnaires by both the interviewer and the observer, and all interviews were audio recorded after the informed consent form was signed. Interviewers used a Microsoft Excel template for data input, noting date and time, overall duration of the interview, the duration of the survey module only, responses, and observations (verbal and nonverbal cues) about respondent response and comprehension. Audio recordings were reviewed in case of conflicting or unclear recorded responses or to extract exact quotes.

### Quantitative pilot

The results of the cognitive testing were used to finalize 2 versions of the questionnaire, with open-ended or closed-ended questions for 4 questions: DGLV, other vegetables, other fruits, and deep-fried foods. The 2 versions had minor differences in the question formulation for eggs and starchy staple foods. (The starchy staple questions were omitted in Form B, due to unresolved debate about whether these questions were necessary, or whether it would be better to save time and assume universal consumption of starchy staples.) They were tested in a quantitative pilot to test whether there were differences between the closed-ended and open-ended approaches and generally to pilot the questionnaire within a multitopic survey setting, where enumerators covered many topics and were not specifically trained on nutrition or food consumption.

The 2 versions were piloted in July–August 2018 in a nationally representative sample of 1000 respondents in Brazil within the Gallup World Poll, a multitopic survey about well-being. Version A and version B were randomly assigned by the computer programming to the respondents, and 519 respondents completed version A and 481 respondents version B. The population sampling included all noninstitutionalized national population 15 y and older, based on the National Statistic Office (Instituto Brasileiro de Geografia e Estatística) 2010 Census. The only geographic exclusions were indigenous lands where interviewers are not allowed to survey. Survey weights were applied to ensure that the sample is representative of the adult population of Brazil.

Differences between version A and version B responses for food groups and diet quality indicators were computed after applying survey weights. A Bonferroni correction for multiple comparisons was applied, so that a significance level of *P* = (0.05/23) = 0.002 would need to be observed for a difference to be considered significant. However, differences of *P* values ≤0.05 are reported as notable.

Indicators of diet quality derived from food groups included the MDD-W; the dietary diversity score for the total population aged 15 and older, calculated as a numeric interval score (0–10) derived from the same 10 food groups of the MDD-W; zero fruits and vegetables, calculated as the percentage who consumed none of all fruit and vegetable categories listed [25]; and β versions of the Global Dietary Recommendations score and its subcomponents, NCD-Protect and NCD-Risk [26].

Survey supervisors and enumerators shared observations from training and fielding the diet quality questionnaire, in comparison with the other survey modules implemented in the Gallup World Poll in Brazil.

## Results

### Pretesting

In the pretest phase to design the formal questionnaires for cognitive testing, several open-ended questions were poorly understood by all or nearly all pretest subjects; these were dropped from the draft questionnaires. Remaining food groups were retained for cognitive testing and piloting, including DGLVs, other vegetables, and other fruits. Table 1 describes observations from pretesting each question.

**TABLE 1:** Open-ended questions pretested.

Pretest question	Issues observed
Any foods made from grains, like bread, rice, or pasta?	In trying to think of other foods that fit this category, the first 3 pretest subjects asked clarifying questions about what should be included, citing other foods made with flour, such as cakes, cookies, and snacks (eg, pretzels and chips). This category refers to foods eaten as staple grains (i.e., the basis of a meal) and not sweets or salty snacks. Nonverbal cues also revealed subjects struggling to decide what they should report, signaling cognitive burden. Result: Open-ended form dropped.
Any roots or vegetables that are orange colored inside, like carrot, pumpkin, orange squash, or sweet potato?	None of the pretest subjects could think of any other orange vegetables popular in these contexts beyond the items listed. Neither could the researchers. Therefore, asking an open-ended question seemed unnecessary. Some questioned what was meant by the term “roots.” Result: Open-ended form dropped.
Any white roots and tubers or plantains, like potatoes or cassava?	Only 1 pretest subject understood the term tuber. One asked if ginger counted as a white root. This question refers to items consumed as starchy staple foods and is not intended to capture ginger or other white roots (eg, ginseng) not used as staple foods. Two respondents asked whether potato chips or French fries should be reported. Result: Open-ended form dropped. In cognitive testing, test a question with the wording, “potato, not including French fries or potato chips.”
Any DGLVs, such as broccoli, spinach, or kale?	Some pretest subjects cited other vegetables that correctly belonged in the group, such as arugula; some cited other vegetables that did not belong: asparagus and iceberg lettuce (as part of a hamburger), which is a variety that is not dark green and does not belong in the category. A few subjects were confused as to why broccoli was presented as an example of a leafy vegetable, which disrupted their confidence in what the category was intended to mean. Result: Open-ended form retained for cognitive testing, but wording flipped to address the concern about broccoli not being a leaf: Broccoli, spinach, kale, or other DGLVs?
Any fruits that are dark orange or yellow inside, like papaya or mango?	Several pretest subjects answered yes to the question because they had consumed oranges. Oranges, despite being orange inside, do not count in this food group because they are not rich in provitamin A. In both pretest countries, oranges are more commonly consumed than papaya or mango. Therefore, it was immediately apparent that this question would likely result in more incorrect answers than correct answers. Result: Open-ended form dropped.
Any other fruits, like apples, oranges, or bananas?	Pretest subjects generally understood this question and responses of items consumed were either those listed or others that fit in the category. One subject realized after the interview that they had answered incorrectly because they did not think of pineapple or avocado when answering, both of which they had consumed but were not listed. They said listing more items would have been helpful to reduce the burden of thinking about other items. Result: Open-ended form retained for cognitive testing. Test shorter or longer lists of examples.
Any other vegetables, like tomato, green beans, or pepper?	Pretest subjects generally understood this question and responses of items consumed were either those listed or others that fit in the category. As with the fruit question, a respondent noted that additional examples would be helpful. Result: Open-ended form retained for cognitive testing. Test shorter or longer lists of examples.
Any eggs?	This was a closed-ended question but is reported here because 2 pretest subjects hesitated and said they were thinking of foods that might have had eggs as an ingredient, such as waffles or cake. (These items should not be included). Result: For cognitive testing, retained this question and also designed a variant specifying “eggs, such as boiled, fried, or in an omelet.”
Any nuts or seeds, such as peanuts, peanut butter, or cashews?	Responses of items consumed were only those listed, so there was no evidence about whether additional responses would belong. Nonverbal cues revealed apparent ease in answering this question. Result: Open-ended form retained for cognitive testing.

Questions about meat and dairy were subdivided into smaller groups/items than those listed in the MDD-W model questionnaire [8], including processed meat, unprocessed red meat (beef, pork, or lamb), poultry (chicken or turkey), cheese or yogurt, and milk. Therefore, open-ended questions were not tested for these food groups.

In the introduction, subjects were observed to be turning inward to recall “yesterday.” Of the first pretest subjects, >1 was observed talking to themselves as they were remembering, describing where they were, and whom they were with throughout the day. The most salient feature of their ability to remember what they ate seemed to be remembering where they were. The conclusion from the first pretest subjects was to incorporate probes in the introduction about eating location. Later pretest subjects listened to both the short and long versions and were asked which one they preferred. Most said the structured (longer) introduction was more helpful for recalling. One person said that with the shorter one, they did not really focus on each distinct eating occasion yesterday. After the introduction, however, no one said it was difficult to recall what they ate yesterday. The entire module, including the introduction, lasted on average 4.5 min, justifying the brief but thorough time spent on the introduction.

### Cognitive testing

The duration of the total cognitive interview was 20 min on average (range: 5–40 min), and the average duration of the dietquality survey module alone was 4 min, 40 s (range: 2–10 min). Characteristics of respondents to the cognitive interviews are tabulated in Table 2.

**TABLE 2:** Demographic data for cognitive interview respondents.

	All (*N*)	Version A (*n*)	Version B (*n*)
Sample	83	44	39
Nationality			
Brazil	46	22	24
United States	12	6	6
China	13	10	3
Iran	6	3	3
Egypt	6	3	3
Gender			
Female	45	23	22
Male	38	21	17
Education			
University degree	40	23	17
No university degree	41	20	21
Missing	2	1	1
Age (y), mean (range)	41 (19–87)	40	41

In response to the question, “Overall, how easy or difficult were the questions to answer?”, nearly all respondents (94%) reported that the questionnaire was easy. When asked, “Do you think some people may have trouble answering any of these questions?”, 36% said yes, citing difficulties elderly people might face with memory issues, those who ate a lot the previous day, and those speaking with different dialects. The latter highlights using language and terms people understand: “some people may have trouble answering questions because the dialect can make them a little hard to understand.” Another respondent said that even for a fluent person, they might not be familiar with all foods. Two others said that people may not remember all the items they ate, “the categories need to be described more.” Others thought elderly people may have trouble answering the questions if there were too many examples and they may have difficulty in memorizing the examples. Finally, “some people may believe it’s hard because they don’t think about what they eat, and eat automatically.” When asked, “Did you feel as if you ‘should’ say yes to some questions, and no to others? If yes, Why?”, 4 respondents said they were self-conscious about what they ate but still told the truth. Everyone else said no.

In response to the question, “Were some questions more difficult than others?”, 15% said yes. One respondent said the most difficult moment in the questionnaire was when they were asked to recall what had been mentioned in previous questions: “Beef or pork, not including the forms previously mentioned.” One respondent of the open-ended questions said for some questions it was “Difficult to understand types of food; categories might not match what people expect.” Three respondents said the ones with longer lists were harder: “as you were reading through that I was thinking about what I was eating, I wouldn’t say it was more difficult but maybe a tiny bit more difficult.” Another said it was harder to recall snack foods because one eats them inattentively.

When asked, “What can we do to make the questionnaire easier to understand?” some said to include more instructions in the introduction on how to respond, including to “Make sure we know there’s no right or wrong answers.” A few respondents said their diet the previous day was not typical and would need reassurance that they should report the unusual day rather than a typical day. One Chinese respondent said to add more items to capture variety in the Chinese diet. Another said “Maybe there is too long of a list but I think it’s good to give more options, in which case just repeating the question is sufficient.” Another suggested written questions may be easier. One said, “Breakfast was hard because I’ve been traveling and I was in a different place yesterday,” underscoring the value of asking people to recall where they were throughout the day in the introduction. There was also a suggestion to break up the content into smaller groupings, suggesting a need for stem questions. One respondent said it would help to speak slowly.

#### Recall period

All respondents said they could remember their food consumption on the previous day, and when asked to describe the recall period in their own words, 100% reported yesterday. Typically, the first question elicited a moment of pause by the respondents as they seemed to need a few extra moments to reflect on the day before. When asked, “Was it difficult to think of yesterday, as opposed to today, or as opposed to your ‘typical habits’?”, most (85%) found it easy to think of the previous day though a few (4) had difficulty differentiating from the present day: “Memories of other days filter in.” Four other respondents talked about yesterday being atypical; 1 said they “felt limited by just thinking about yesterday, because what I ate yesterday was very unusual compared to other days.” However, a respondent said the wording helped “because you said ‘yesterday’ over and over again so it was easy to think about what I ate,” which shows that the stem questions were helpful because they remind respondents about the period.

#### Mixed dishes

Almost everyone reported that they did consider ingredients in mixed dishes as well as foods eaten alone. When respondents were probed about which foods they consumed when answering yes to a question, the foods reported were often part of mixed dishes. Some people noted considering green vegetables as part of a stew or lettuce as part of a hamburger. Those who ate meals with a variety of ingredients such as a salad or a burrito paused to consider all ingredients. We were especially concerned about reporting milk, which in these contexts is often taken with coffee or other milk-based drinks (e.g., *vitaminas* in Brazil) in significant amounts. When probed, all but 1 respondent did consider milk in tea and coffee, and for most, that is the item they reported consuming. Of 354 yes responses to items where mixed dishes were possible, 206 of the items (58%) were reported as consumed within a mixed dish. The proportion consumed in mixed dishes was highest for DGLV (74%) and poultry (81%) and lowest for white roots like potato (41%) and nuts (26%).

#### Open-ended questions

DGLVs were not understood in the same way by all respondents. In Brazil, a correct response for the open-ended question on DGLV was escarole; incorrect responses were cabbage and lettuce. Two Egyptian respondents included green onion and cucumber. Iranian respondents included celery, cauliflower, lettuce, and coleslaw. Chinese respondents were confused by the term DGLVs in the open-ended question. Chinese respondents said they think of bok choy and cabbage in the same grouping, and therefore listing round cabbage under other vegetables and bok choy under DGLV would be confusing to many Chinese respondents when asking them to categorize similar items into each category.

“Other vegetables” was also not universally understood. In Brazil, 2 people incorrectly reported having eaten potato as a positive response to the open-ended question about vegetables. In the United States, some respondents reported DGLVs, avocado, and onion (which is often used in small amounts for flavor and is therefore excluded). “Other fruits” seemed better understood, but 1 person reported lemon as a positive response, which was not desired because it is consumed in small quantities as a flavoring.

Table 3 summarizes the results from the open-ended questions. When respondents answered yes to an open-ended question, the interview team followed up to ask what item(s) they actually ate. The majority of respondents (70%–80%) reported consuming only foods among the 3–5 examples given, and no others (Table 3). When we asked respondents what other items would belong in the food group, only 50% of respondents were able to suggest any items for DGLV and only 35% for nuts and seeds; the rest said they could not think of any other examples apart from those given. Of the respondents who reported consuming items other than the examples, for DGLV, most reported ≥1 item that did not belong (6/8 people), and for other vegetables, all (6/6 people) reported an item that did not belong. There was less misclassification for other fruits and nuts and seeds, where only 1 of the 8 respondents reported consuming an unlisted item that did not belong for other fruits; only 3 reported another nut or seed, all of which belonged. However, when asked to suggest additional items that participants think belong in the nuts and seeds category, 38% listed an item that did not belong. Items that were correctly and incorrectly classified are listed in Table 3. Of all items participants reported consuming other than the examples listed, 49% of them were misclassified: higher for DGLV and other vegetables and lower for other fruits, nuts, and seeds.

**TABLE 3:** Results from testing the open-ended forms of questions.

Food group	DGLV	Other vegetables	Other fruits	Nuts and seeds
Percentage of respondents who reported consuming only the example foods given	70 (19/27 yes responses)	79 (23/29 yes responses)	74 (23/31 yes responses)	80 (12/15 yes responses)
Apart from the examples given, percentage of items reported as consumed that do not belong in the food group	64 (7/11 items)	82 (9/11 items)	10 (1/10 items)	0 (0/3 items)
Percentage of respondents who reported consuming ≥1 food that does not belong in the food group	75 (6/8 respondents)	100 (6/6 respondents)	13 (1/8 respondents)	0 (0/3 respondents)
No. of 83 respondents who were correctly classified as yes based on items consumed other than the 3–5 examples listed^1^	1	0	3	1
No. of 83 respondents who were incorrectly classified as yes based on items consumed other than the 3–5 examples listed	2	3	1	0
Percentage of respondents who were able to think of any other items that might be in the food group	50 (40/80, 3 missing)	Not asked	Not asked	35 (29/82, 1 missing)
Percentage of respondents who listed ≥1 food that do not belong in the food group, when suggesting other items they believe belong in the food group	50 (20/40)	NA	NA	38 (11/29)
Items reported that belong: consumed (and suggested)	Arugula, broccoli rabe, escarole (beetroot greens, turnip greens, watercress, kale, and pea leaves)	Heart of palm	Avocado, lychee, longan, pear, raisins, and dried fruits	Cashews, mixed nuts, sunflower seeds (pumpkin seeds, melon seeds, pistachios, walnuts, hazelnuts, and macadamia)
Items reported that do not belong: consumed (and suggested)	Basil, cucumber, green onion (cabbage, lettuce, coleslaw, cauliflower, celery, sweet potato, and tomato, carrot)	Avocado, broccoli, kale, onion	Lemon	Dried fruits, cereal grains such as rye, flax seeds (an ingredient used in small amounts), and nutmeg

Abbreviations: DGLV, dark green leafy vegetable.

1 The list tested included only 3–5 example items in each food group. In current practice, 14 example items are used in closed-ended questions for DGLV, other vegetables, and other fruits, and 7 example items are used for nuts and seeds. Using these current questionnaires for Brazil, the United States, China, Egypt, and Iran (available at dietquality.org/tools), no respondents would have been misclassified due to missing example items.

The cognitive testing also revealed that many respondents could not think of other items beyond those listed; only half of respondents could think of additional other DGLVs beyond the example items and 35% could think of other nuts or seeds. Many of the additional items respondents thought of did not belong in the group (Table 3). Misclassification was also present in other food groups with open-ended questions. One respondent reported cheese bread (pão de queijo) as belonging in the deep-fried foods open-ended question; this is incorrect because it is not deep fried, although is a popular snack food, and it is understandable that respondents think of it as in the same category as deep-fried foods. Several others reported deep-fried foods, nuts, crackers, and cookies as belonging to the packaged salty snack category, which is meant to only include chips and puffs.

Despite clear evidence of misclassification in respondents’ cognitive processes in responding to open-ended questions, few people would have been misclassified as yes or no for the food group, because many people also consumed the example items listed. In total, 6 of the 83 would have been misclassified as false positives in open-ended questions where incorrect items were reported, and 5 of the 83 would have been misclassified as false negatives by asking closed-ended questions containing only 3–5 items (Table 3). These results suggest that >3–5 items are needed to capture diverse food groups such as vegetables, fruits, nuts, and seeds.

#### Closed-ended questions

Overall, respondents had no clear preference for shorter lists (3–5 example items) or longer lists (7 items). Many were indifferent, and for those who stated a preference, half preferred the short version and half preferred the long version in all contexts.

Some respondents stated the shorter version was better because it was quick and to the point. Many who preferred the shorter version seemed to show a preference for speed over completeness/accuracy (“Less options, simpler”; “The second version took too long”). One respondent indicated preference for “the shorter list because by the time you get to the end of the list, it’s hard to remember the beginning.” Another said “A vegetable is a vegetable, no need for a long list”—a sentiment contradicted by the finding that 100% of respondents who reported additional vegetables beyond the examples given included an item inconsistent with the researchers’ categorization.

Other respondents preferred the longer version as it helped them remember other foods consumed the previous day. Several people who preferred the longer version said that it was easier to understand what we were asking about with more specific examples and that they (or others) might not think of further examples if they were not spoken aloud (“There’s more variety and it’s easier to recall”; “With more options people may identify a food they [could not think of] the name”; “I preferred more examples because many people don’t understand food groups. So more examples make it more specific what you are asking.”) Another respondent said, “fewer examples is harder because you’re not assisting the person at all,” indicating that the cognitive load was high trying to come up with other similar foods that belong in the food group. In the United States, 1 respondent said they preferred the longer fruit question “because I don’t think I would have lumped avocados in with that.”

#### Introduction and question stems

The importance of a clear introduction was shown to be crucial for retrieval, and guiding the respondent through the previous day to facilitate recall of food consumption was particularly helpful based on nonverbal cues and speed and fluency of response at the beginning of the questionnaire.

Question stems were helpful (e.g., “Yesterday, did you eat any of the following fruits:”). Tested with, and without, and enumerator feedback indicated that it was easier for people to follow the questionnaire with stems included. Without preceding the sweets questions by saying “did you eat any of the following sweet foods?” some people reported salty biscuits. Furthermore, the stems serve to remind the respondent of the recall period (yesterday).

#### Asking respondents to exclude items from consideration

Two questions included phrasing asking respondents to exclude items from consideration. We did not expect this wording to be problematic. However, the instruction to exclude foods was noted as particularly difficult, and in several instances, respondents answered the questions in the opposite way intended, reporting exactly the items they were instructed to exclude. For the question, “Potatoes, not including French fries or potato chips?”, 4 respondents reported eating French fries. It is probable that respondents were not listening carefully, and were affected by the psychological principle of “recency” – the tendency to recall the last item on a list more than the words in the middle [27]. This makes sense, because the question formulation deviated from the question formulation that respondents had become accustomed to in the interviews. As noted earlier, the question asking respondents to exclude previously cited meats from consideration was also singled out as a difficult question.

#### Terminology and translations

The questionnaire wording sometimes uses “umbrella terms” including beans, fish, and cheese—each of which capture many species or varieties. Based on cognitive feedback as well as examining the items actually reported, these terms presented no problems in understanding. Although eggs was flagged by 2 pretest respondents as potentially confusing, it presented no difficulty in any context in the cognitive testing; no one answered about eggs as a culinary ingredient, everyone answered about eggs as a whole food.

On the contrary, some umbrella terms had specific linguistic/translation issues. In Brazil, the questionnaire used a generic term for “cold cuts” (frios), which was intended to capture only processed meat but whose Portuguese translation also includes cheese. In the final version, therefore, no generic term was used and only specific sentinel items were listed. Soda/soft drinks generally worked well when translated across languages, but the Farsi translation meant carbonated beverage, so some Iranian respondents included plain seltzer in their responses, which was not intended. (In the United States, pretesting had revealed that soft drinks was not universally understood, so soda or pop was the term used in the survey instead, which presented no problems in cognitive testing.) This finding signaled that care needs to be taken in selecting the right term in the final translation, including >1 popular term for the same item if needed and not just the official or formal English term. Fast food was understood inconsistently by respondents. To Chinese and Iranian respondents, the term meant simply food that can literally be picked up quickly. Their responses included the take-out foods such as pizza, hamburgers, Chinese food, and any food from the food court including sushi. The term also was not understood in Brazil where the English term fast food was used, as there was no Portuguese translation. Based on these findings, we abandoned the use of the term fast food altogether, seeing that there was no universal understanding of its meaning. Instead, the final questionnaire implemented in the quantitative pilot asked about food consumed from “any place like [list of well-known fast food brands]”. It was easier for respondents to answer the questions about soft drinks and fast food places when given examples of brand-name products/places.

### Quantitative pilot

Sample characteristics between version A and version B showed no statistically significant differences in age, gender, education, region, or urbanicity. Sample characteristics are shown in Supplemental Table 1. Both module versions took approximately three and a half minutes to administer in the Gallup survey (mean of 3:29 for version A, and 3:25 for version B).

The Gallup enumerators did not report any problems or difficulties with this module; their feedback was that it was at least the same level of feasibility (or easier) than World Poll modules on other topics.

Prevalence of consumption and confidence intervals for each food group are shown in Supplemental Figure 1 and Supplemental Table 2. Between version A and version B, 3 three of the 26 questions resulted in notable differences (*P* < 0.05), although none of these remained statistically significant after applying a Bonferroni adjustment for multiple comparisons: DGLV (17.5% from closed-ended and 26.6% from open-ended questions; *P* = 0.04), fish or seafood (13.1% compared with 6.9% from respondents of version A compared with those of version B; *P* < 0.01), and instant noodles, frozen lasagne, or instant soup (10.9% compared with 15.9% from respondents of version A compared with those of version B; *P* = 0.02). The latter 2 food groups were exactly the same question in version A and B, so there is no explanation for the differences in those food groups (illustrating why we apply the Bonferroni adjustment for multiple comparisons). There were no notable differences between closed-ended and open-ended questions for the other 4 food groups that were tested: other vegetables (57% closed compared with 63% open; *P* = 0.10), other fruits (64% closed compared with 63% open; *P* = 0.96), deep-fried foods (20% closed compared with 21% open; *P* = 0.62), and eggs (44% in each form; *P* = 0.29). There were no statistically significant differences in diet quality indicators calculated from version A compared with those from B (Table 4).

**TABLE 4:** Indicators calculated from versions A and B with 95% **confidence intervals**.

Indicator	Version A (closed-ended)	Version B (open-ended)
Ate a starchy staple food yesterday	96 (92.3–97.9)	Data not collected, assumed 100
MDD-W	75.7 (70.2–80.5)	77.7 (72.0–82.5)
DDS	5.8 (5.6–6.0)	5.9 (5.7–6.0)
β-Version of GDR score (range: 0–15)^1^	7.2 (7.0–7.5)	7.1 (6.9–7.3)
β-Version of NCD-Protect (range: 0–9)^1^	3.4 (3.2–3.7)	3.4 (3.3–3.6)
β-Version of NCD-Risk (range: 0–6)^1^	2.2 (2.0–2.4)	2.4 (2.2–2.5)
Zero fruits and vegetables	15.1 (11.2–19.9)	9.7 (7.1–13.0)

Abbreviations: DDS, dietary diversity score; GDR, Global Dietary Recommendations; MDD-W, Minimum Dietary Diversity for Women; NCD, noncommunicable disease.

1 This pilot data collection predated the completion of the indicator validation, and food groupings in the pilot were slightly different; therefore β versions were calculated with the available food groups. NCD-Protect-β was calculated from 9 healthy food groups: 3 fruit groups (vitamin A–rich fruit, citrus, and other or red/blue), 3 vegetable groups (dark green leafy vegetable, vitamin A–rich vegetables, and other vegetables), legumes, whole grains, nuts, and seeds. NCD-Risk-β was calculated from 6 food groups to limit or avoid: soft drinks, baked and other sweets, processed meat, red meat, deep-fried foods, fast food. GDR score-β was calculated as NCD-Protect-β minus NCD-Risk-β. These food groupings changed slightly after the completion of this pilot study and the indicator validation study [26].

## Discussion

The results show overall ease and cognitive validity of the questionnaire regarding comprehension (questions were well understood), retrieval (respondents reported being able to recall yesterday, especially with taking time to recall in the introduction), and response (the respondents were able to provide yes or no answers). The pretest revealed some immediate issues with comprehension of technical terms (e.g., tubers), and the cognitive test showed evidence of lack of comprehension other terms including fast food, packaged snacks, and deep-fried foods. Open-ended questions had problems with judgment because respondents could not necessarily provide consistent answers to which food items count within a given food group.

Respondents in all settings and languages did not always categorize foods in the same way. For some food groups, the majority of respondents could not think of other foods belonging to the food group, gave examples inconsistent with the intended grouping, or could not understand what the food group represented. These results illustrate how mental categorizations of foods by respondents are often reasonable, but differ in meaningful ways from the categories nutritionists use for diet indicators. A cognitive testing process does not seek to identify representative statistics on the frequency of misclassification, but it establishes the existence of uncertainty and resulting misclassification, which even in a small sample in each setting, was widespread.

Given the observed lack of cognitive validity of open-ended questions in the cognitive testing, it is surprising that the quantitative pilot test did not show larger differences between open-ended and closed-ended questions, nor differences between diet indicators. It is possible that the 2 opposing types of misclassification in the open-ended questions (overreporting based on incorrect items and underreporting based on lack of probing) may have canceled each other out at population level.

Although there were not quantitative differences between the 2 approaches, based on the strong evidence of misclassification and cognitive burden from cognitive testing, we conclude that open-ended questions lacked cognitive validity. Closed-ended questions were well understood and, therefore, are preferred. Unlike open-ended questions, closed-ended questions do not state the food group name, so stem questions appeared useful to help respondents more easily follow the type of questions being asked (e.g., “Yesterday, did you eat any of the following vegetables: …?”). The results also show that closed-ended questions for the most diverse food groups (fruits, vegetables, DGLV, fried foods, and sweet foods) need to have >3–5 sentinel foods. Listing too few items risks a high proportion of false negatives, especially when considering regional diversity in large food groups such as other fruits and other vegetables. We tested ≤7 items, which has been suggested as the maximum for cognitive retention [28]. For diverse food groups, if >7 items are needed, an additional question could be added for the same food group, and the results combined in the analysis. In principle, this solution would solve the problem of obtaining accurate data for large food groups while still using short questions for cognitive validity. Each additional question and item may add to respondent fatigue, especially relatively longer questions. When many sentinel foods are required to capture large food groups, it is unclear whether fatigue is more effectively reduced by subdividing into as few questions as possible (≤7 items per question) or into more questions that have fewer items each (of 3–5 items per question). What is clear from the results of this study, however, is that the trade-off between accuracy and fatigue cannot be solved with open-ended questions, with the hope that respondents will be able to think of other items that belong with the examples given and consistently categorize those items. The evidence does not support that approach.

Limitations of the study include that we suspected some underreporting of items that were not listed as example foods but could not test whether that was occurring without an objective standard of comparison such as a weighed food record. Whether closed-ended questions composed of sentinel foods sufficiently capture a food group is shown in other research [29], but we could not assess the possibility of telescoping, that is, with more items listed, participants may be more likely to say yes even if they did not consume the specific items in the previous day. Uyar et al. [30] showed that the Diet Quality Questionnaire (DQQ) composed of closed-ended questions using sentinel foods generally produced similar prevalence of consumption to quantitative 24-h recalls, but differences tended toward higher prevalences compared with the reference method, not lower prevalences (which would have occurred if sentinel foods were missing). Comparisons with 24-h recalls also have limitations because underreporting is a well-documented problem in the 24-h recalls and varies by type of food [[31], [32], [33]]. Observational studies can help answer if closed-ended questions may be prone to overrerporting; one such study reported close similarity of the DQQ for infants and young children to observed consumption in 1 country, and a tendency toward overreporting of both the DQQ and 24-h open recall in a second country [34].

While other studies can compare the closed-ended approach to a reference method, the evidence from this study demonstrates poor cognitive validity of the open-ended approach, and apparent cognitive validity of the closed-ended sentinel food method, and has led to widespread use of the closed-ended approach in practice. Based partly on these results, the FAO MDD-W guide update (2021) was revised to encourage users to use sentinel foods for the list-based method. Questionnaires comprising closed-ended questions using sentinel foods have been adapted for 140 countries [23,35]. Gallup World Poll has implemented adapted questionnaires in 94 countries based on these results from 2021 to 2024 [35]. The Demographic and Health Surveys use these question adaptations for women and children, having also supported cognitive testing of the approach [19,36]. These global surveys are the first to capture the MDD-W indicator using a consistent approach that has been tested for cognitive validity.

## Author contributions

The authors’ responsibilities were as follows—AH, PDR, AR: designed the research; AH, IS, DO, PDR: conducted the research and analyzed data; when data were collected, AH was an affiliate of Columbia University and IS and DO were doctoral candidates at University of São Paulo and Teachers College, Columbia, respectively; AH, IS, DO: wrote the paper; AH: had primary responsibility for final content; and all authors: have read and approved the final manuscript.

## Funding

This work was supported by the Swiss Agency for Development and Cooperation (SDC) for financing the cognitive testing and by the Government of Canada via BPNR at the Global Alliance for Improved Nutrition (GAIN) for financing the quantitative pilot, and by GAIN for managing reporting of the process and results. The supporting sources had no involvement in study design; collection, analysis, and interpretation of data; writing of the report; or restrictions regarding the submission of the report for publication.

## Data availability

Data described in the manuscript, code book, and analytic code will be made available upon request pending application and ethics approval, and pending payment in the case of quantitative data from the 2018 Gallup World Poll.

## Conflict of interest

All authors report no conflicts of interest.
