# Supplementary material for: You Say Potato, I Say Vegetable; You Say Tomato, I Say Fruit: Cognitive Validity of Food Group–Based Dietary Recall Questions
Source: Curr Dev Nutr. 2024 Nov 4;9(Suppl 1):104502. doi: 10.1016/j.cdnut.2024.104502 (PMC12125696; doi:10.1016/j.cdnut.2024.104502)

Herforth, A.W., Sattamini, I. F., Olarte, D.A., Diego-Rosell, P., Rzepa, A. You say potato, I say vegetable; you say tomato, I say fruit: Cognitive validity of food group-based dietary recall questions. Current Developments in Nutrition (2024), 10452.

[*https://doi.org/10.1016/j.cdnut.2024.104502*](https://doi.org/10.1016/j.cdnut.2024.104502)

# Supplementary Material

Table of Contents

[Supplementary Material 1](#_Toc179549551)

[Model questionnaire and introductions used in pretesting 2](#_Toc179549552)

[Cognitive testing interview guide for enumerators 4](#_Toc179549553)

[Acculturation screener for interviews conducted in New York City 6](#_Toc179549554)

[Cognitive Testing: Summary of issues tested and differences between Questionnaire Version A and B 7](#_Toc179549555)

[Cognitive Testing Questionnaire A template for New York City 9](#_Toc179549556)

[Cognitive Testing Questionnaire B template for New York City 12](#_Toc179549557)

[Cognitive Testing Questionnaire A implemented in greater São Paulo, Brazil 15](#_Toc179549558)

[Cognitive Testing Questionnaire B implemented in greater São Paulo, Brazil 18](#_Toc179549559)

[Region of origin of cognitive interview respondents 23](#_Toc179549560)

[Cognitive testing responses to qualitative questions 24](#_Toc179549561)

[Quantitative pilot test in the Gallup World Poll: Differences between questionnaire versions A and B implemented in a national sample in Brazil, 2018 25](#_Toc179549562)

[Quantitative pilot test in the Gallup World Poll: Questionnaire version A and B as implemented in the Gallup World Poll in Brazil, 2018 27](#_Toc179549563)

[Supplementary Table 1. Sample characteristics of the two questionnaire versions implemented in the Gallup pilot test in Brazil, 2018 30](#_Toc179549564)

[Supplementary Table 2. Prevalence levels and confidence intervals of the two questionnaire versions implemented in the Gallup pilot test in Brazil, 2018 31](#_Toc179549565)

[Supplementary Figure 1. Proportion responding “yes” to each question in Form A and B, in increasing order of prevalence 32](#_Toc179549566)

## Model questionnaire and introductions used in pretesting

Food group questions for initial pretesting were taken from the model list-based questionnaire for data collection for the minimum dietary diversity for women (MDD-W) indicator (FAO and FHI360, 2016).


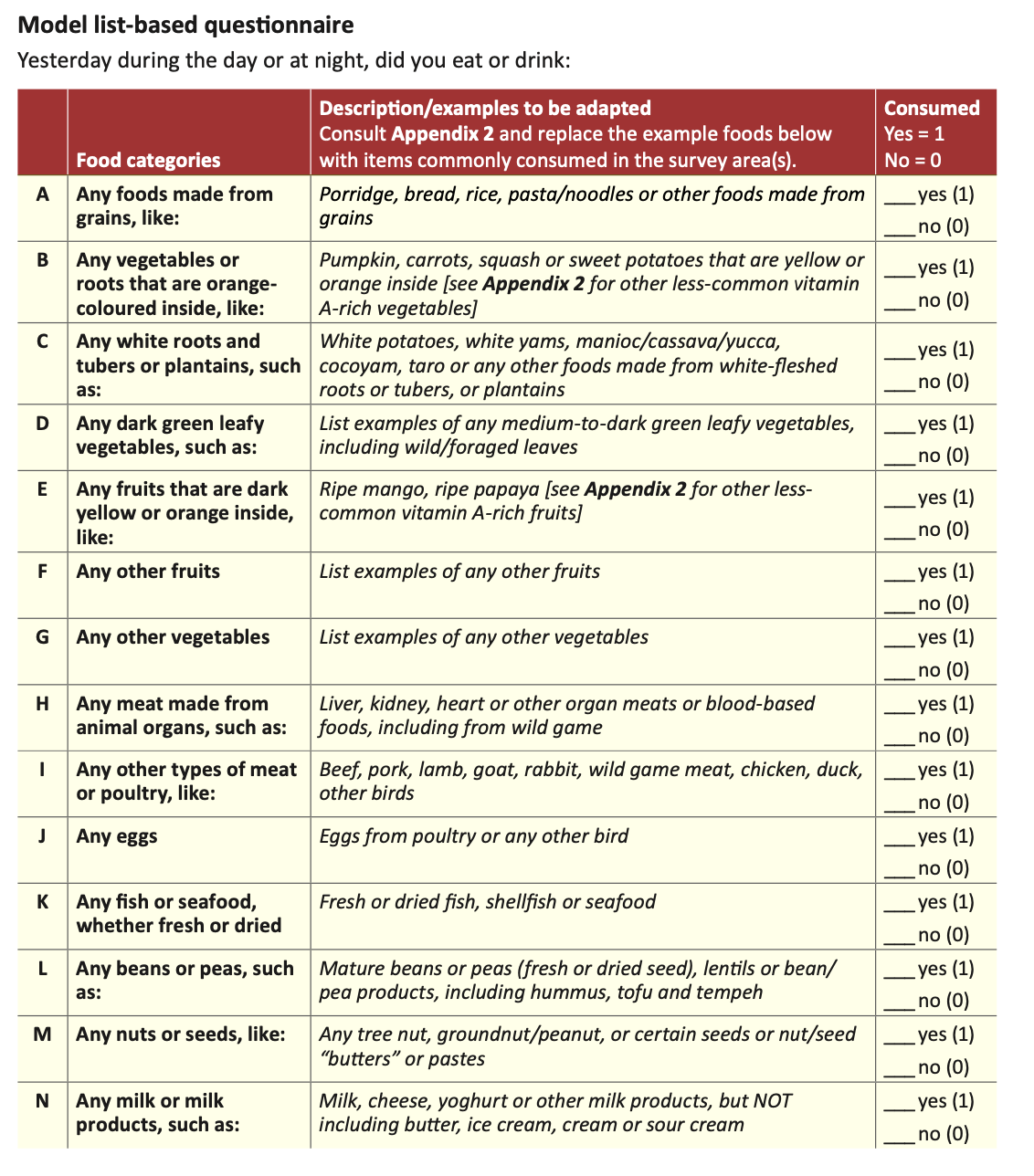


Source: FAO and FHI 360. Minimum dietary diversity for women: A guide to measurement. Rome: FAO; 2016.

Note: We did not test open-ended questions for items H, I, and N (organ meat, meat, and dairy), because we had subdivided those groups a priori (processed meat, unprocessed red meat, poultry, cheese and yogurt, fluid milk) based on considerations around differential dietary impact related to noncommunicable diseases. These were closed-ended because they contained only few items in each setting.

Later pretesting included shorter or longer forms of questions (with a varying number of example items), the results of which were inconclusive. Therefore the number of examples to include in a question was tested in cognitive testing.

Later pretesting also tested two different introductions:

1. “Now, I would like to ask you some questions about the types of food you ate yesterday. Please include any foods you ate for breakfast, lunch, dinner, or as a snack, meals during any time of the day.”
2. “I will be asking you some questions about what you ate yesterday, but first I would like you just to think about yesterday, from the morning until the end of the day. Think about the first thing you ate or drank after you woke up in the morning …(pause a moment)…think about where you were when you any food or drink in the middle of the day …(pause a moment)…and any snacks or drinks you may have had in the morning, between meals …(pause a moment)…Think about where you were when you had an evening meal …(pause a moment)…and any snacks or drinks you may have had in the afternoon, between meals …(pause a moment)…and where you were after dinner, and any food or drink you may have had in the evening...(pause a moment). Now I will ask you about what you had to eat or drink yesterday. When I ask you each question, please consider foods eaten in mixed dishes as well as foods eaten on their own.”

## Cognitive testing interview guide for enumerators

**Gallup Project: Interview Guide**

Each interview will be held by a pair of trained experienced interviewers. Roles are defined as: one interviewer implements the form and the other records and observes.

The interview will be held in three stages:

Introduction: Explain that we are going to ask some questions about food consumption, followed by some additional questions about the interview process. See general instruction below.

*Hello my name is __________, I am a student at Teacher’s College. We are doing a research study on food. Would you have time to answer some questions?*

*Thank the person whether they agree or decline.*

*If agreed, ask their name, and have a brief friendly interaction and continue:*

*“It is a totally voluntary participation, you can interrupt it anytime you like, you don’t have to answer any questions you don’t feel comfort with, all of your personal information is confidential and will not be shared.”*

*NEW YORK ONLY: “I have just a few questions to determine whether to continue the survey. No question has a right or wrong answer and this is not a test in any way. Can I continue?”*

*If agreed, ask the Acculturation Module.*

*🡪 if the person does not qualify, say “Thank you for your time; we don’t need to continue with the other questions but we really appreciate your willingness!”*

*Hand them the consent form to keep.*

*🡪If the person qualifies, say “Thank you, next I have some questions about what you ate yesterday. It won’t take longer than 20-30 minutes. The interview will be recorded only for research purposes, the content will not be shared. Is it ok to record?”*

*Find a place to sit, hand them the Informed Consent Form, and get their signature.*

*Turn on the recorder*

Choose to administer Version A **or** Version B of the questionnaire. Read the introduction, and pause to let the respondent think about what he or she ate yesterday. Go through Pass 1 of the module. Do not modify the wording of the module questions. Then go through Pass 2 of the module, asking the questions as indicated according to yes/no answers.

**Record answers as given, and record any verbal and non-verbal cues that may be of interest on the notes template (hesitation, clarifying questions, among others).**

Ask the additional general questions. Probe as necessary to understand the respondent’s thoughts about the questions.

Finally, ask the demographic questions: age, education, and region of origin. Thank them for their time.

**General Instructions**

**Before the beginning of the cognitive interview:**

1. Share with the respondent a few words about yourself, and that you are a student. Tell them the goal of the survey today is to determine whether the questions on the survey are easy for people to understand. Explain how important the respondent’s help is to ensuring we end up with the right questions.
2. Stress to the respondent that we are not conducting a regular survey, but testing a questionnaire that has questions which may be difficult for some people to answer. We are seeking the respondents’ help in improving our questionnaire.
3. We hope that respondents will answer the questions honestly and they should not hesitate to tell us if they can’t understand something, or if any questions make them uncomfortable.
4. Feel free to say, “I did not write these questions, so don’t worry about hurting my feelings if you criticize them. My job is to find out what isn’t clear and what needs to be fixed.”
5. Be sure to go through the interview at an easy pace. Do not rush the respondent.
6. Probe as often as you need to in order to gain additional information from the respondent.
7. Do NOT explain the questions to respondents. We want to know whether they can understand the questions on their own.

***General probes to use as needed in Pass 2 only:***

*I noticed that you hesitated. Can you tell me what were you thinking?*

*How sure are you of your answer?*

*Tell me more.*

## Acculturation screener for interviews conducted in New York City

This acculturation screener for participation was used in interviews of recent immigrants from China, Egypt, and Iran living in New York City. The example shown is for Chinese immigrants. When used for Egyptian immigrants, the word “Chinese” would be replaced with “Arabic” for language (Q1-4) and “Egyptian” otherwise (Q5-8). When used for Iranian immigrants, the word “Chinese” would be replaced with “Farsi” for language and “Iranian” otherwise. This screener was modified from: *Suinn RM, Ahuna C, Khoo G. The Suinn-Lew Asian Self-Identity Acculturation Scale: Concurrent and Factorial Validation. Educational and Psychological Measurement. SAGE Publications Inc; 1992;52:1041–6.*

**Modified Acculturation Scale**

|  | | | |  |  |  | |  | |  | |  | |
| --- | --- | --- | --- | --- | --- | --- | --- | --- | --- | --- | --- | --- | --- |
| *Instructions: To continue with the interview, the respondent's answers must fall within the highlighted boxes outlined below.* | | | | | | | | | | | | | |
|  |  |  |  |  |  |  | |  | |  | |  | |
|  |  |  |  |  | No | Yes | |  | |  | |  | |
| 1 | **Are you fluent in Chinese?** | | |  |  |  | |  | |  | |  | |
|  |  |  |  | *If no, thank the person and gently end the interview.* | | | | | | | | |  |
|  |  |  |  | *If yes, continue:* | | |  | |  | |  | |  |
|  |  |  |  |  | Only Chinese | Mostly Chinese | | Equally Chinese and English | | Mostly English | | Only English | |
|  |  |  |  |  |  |  | |  | |  | |  | |
| 2 | In general, what language do you read? | | | |  |  | |  | |  | |  | |
| 3 | In general, what language do you think in? | | | |  |  | |  | |  | |  | |
| 4 | What news media do you usually read or watch? | | | |  |  | |  | |  | |  | |
|  |  |  |  |  | Only Chinese | Mostly Chinese | | Equally Chinese and non-Chinese | | Mostly non-Chinese | | Only non-Chinese | |
| 5 | **What kind of food do you usually eat?** | | |  |  |  | |  | |  | |  | |
| 6 | What kind of food do you usually eat at home? | | | |  |  | |  | |  | |  | |
| 7 | What kind of food do you usually away from home? | | | |  |  | |  | |  | |  | |
| 8 | Your close friends are: | |  |  |  |  | |  | |  | |  | |

**If NOT Qualified: Thank you for your time.**

## Cognitive Testing: Summary of issues tested and differences between Questionnaire Version A and B

- 1. For all food groups with a yes answer:
     1. What specific food or foods did you eat?
     2. Were these part of mixed dishes, or eaten individually?
     3. Ask if they remembered the instructions to report as consumed whether the foods were eaten singly or in mixed dishes.
  2. For dairy, regardless of yes or no answer:
     1. If yes: what food or drink did you consume?
     2. If no: Did you think about café con leite, vitaminas, or sweetened milk drinks?
  3. For DGLV, nuts and seeds, salty snacks, industrial meals, fast food place:
     1. What does the term "[dark green leafy vegetables]" mean to you?
     2. Ask respondent to identify other similar foods beyond the 3-5 provided.
  4. For the DGLV, other vegetables, or other fruit groups: Which version of this question is easier for you? *(read 5 example version; then read 3 example version)*
  5. Describe in your own words what the recall period was.
     1. Was it difficult to think of yesterday, as opposed to today, or as opposed to your typical habits?
  6. Overall, how easy or difficult were the questions to answer?
     1. Were some questions more difficult than others?
     2. Do you think some people may have trouble understanding/recalling any of these questions?
     3. *If yes on any specific question:* What can we do to make this question easier to understand?
  7. Why do you think you were being asked about your diets (i.e. do they mention healthy diets, or other reasons they think might be why they were asked to report their diets)
     1. Did you feel as if you “should” say yes to some groups, and no to others? Why?
  8. Do you have any more comments or suggestions on this questionnaire?

| Version A | Version B |
| --- | --- |
| Open-ended questions for DGLV, other vegetables, other fruits, nuts & seeds (using 3-5 examples)  [list the top 3 items], or any other dark green leafy vegetables? | Closed-ended questions for all food groups, using 5-7 examples |
| Stem questions with broad food groups | Generic stem questions (“Yesterday, did you eat any of the following?”) |
| Lists egg preparations | Asks simply about “eggs” |
| Lists brand name examples for packaged salty snacks, sodas, and fast food restaurants | Just lists types of snacks, word “soft drinks” with no examples, and “fast food” with no examples. |

## Cognitive Testing Questionnaire A template for New York City


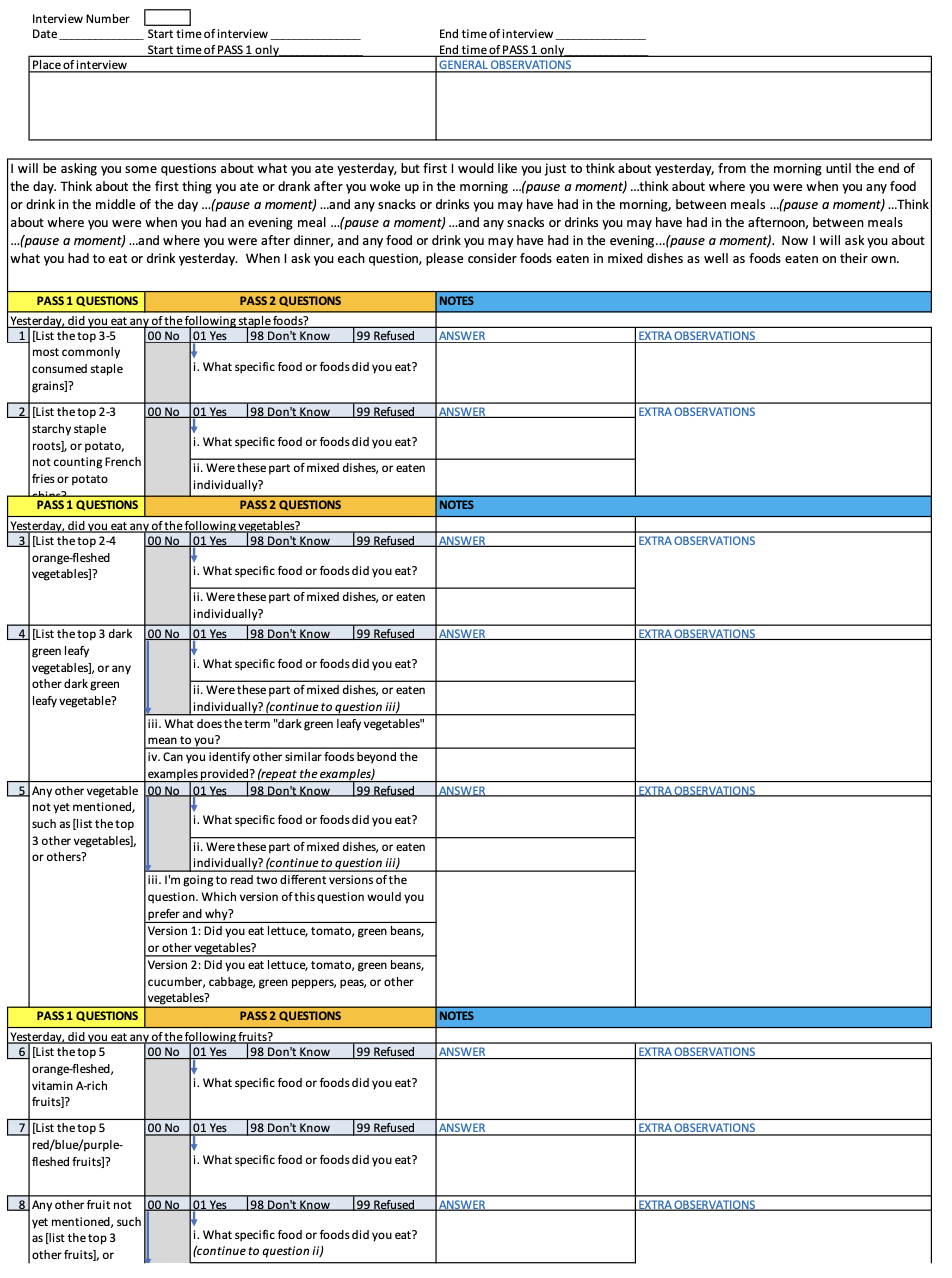


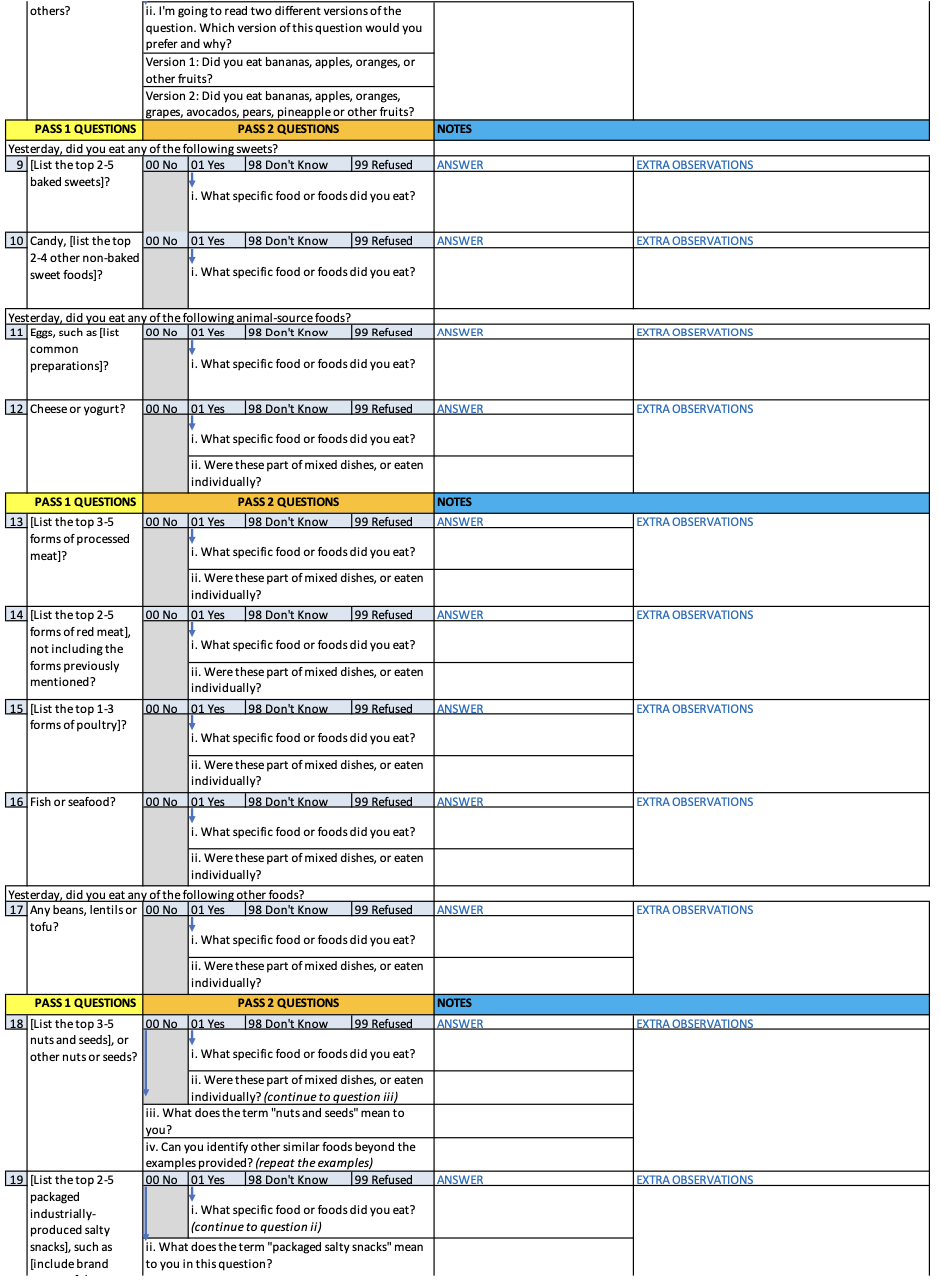


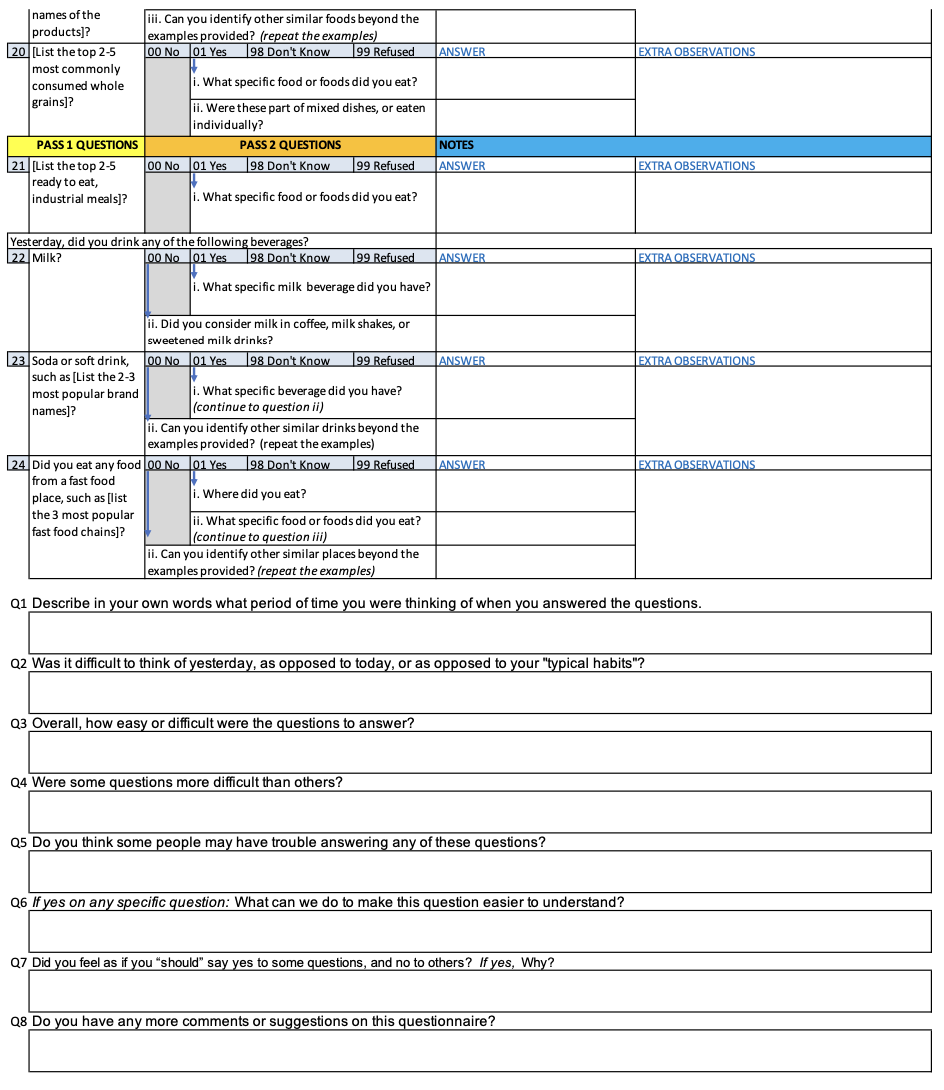


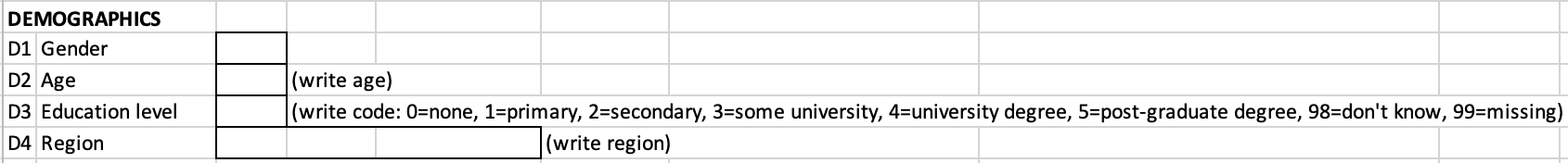


## Cognitive Testing Questionnaire B template for New York City


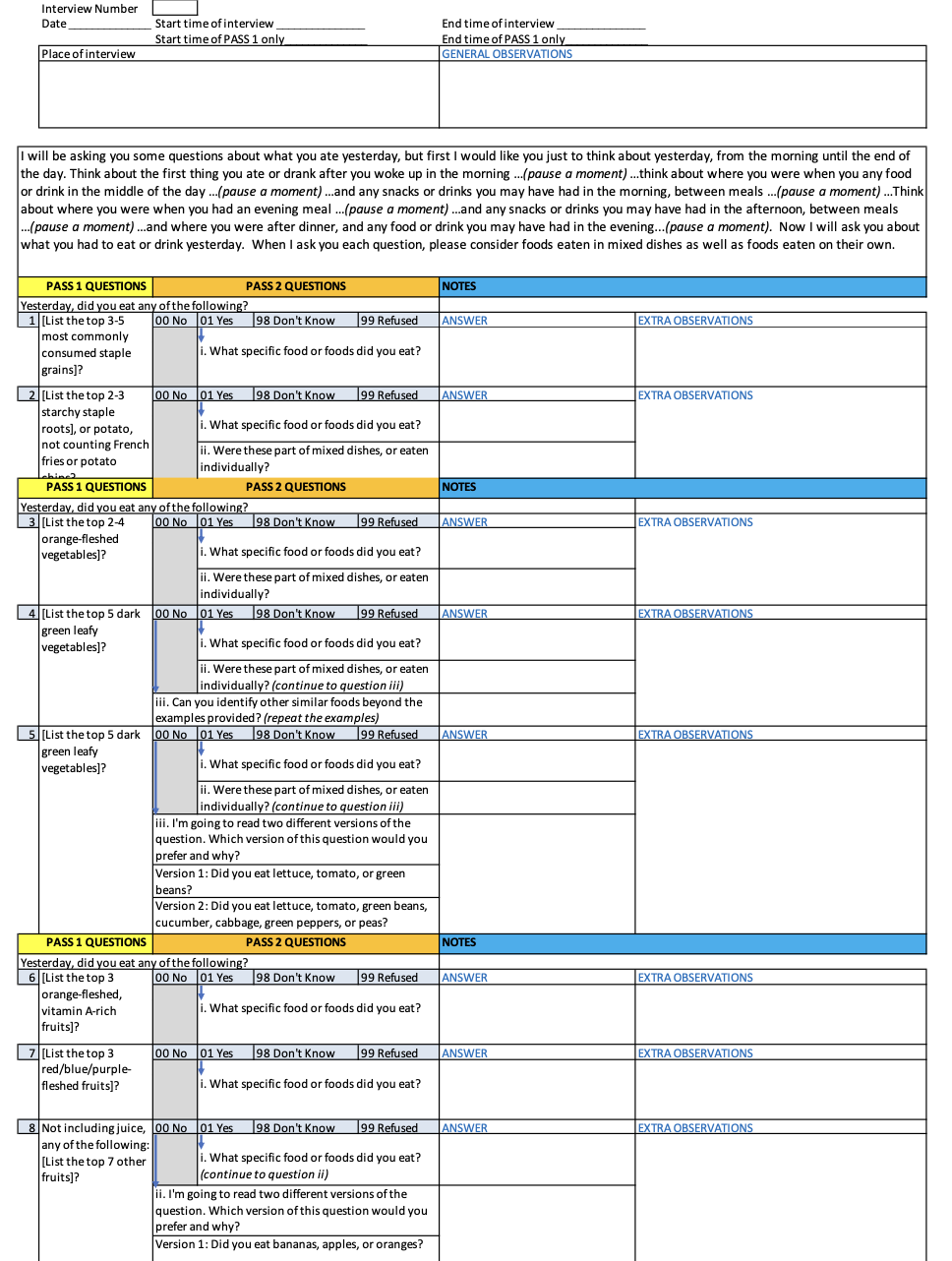


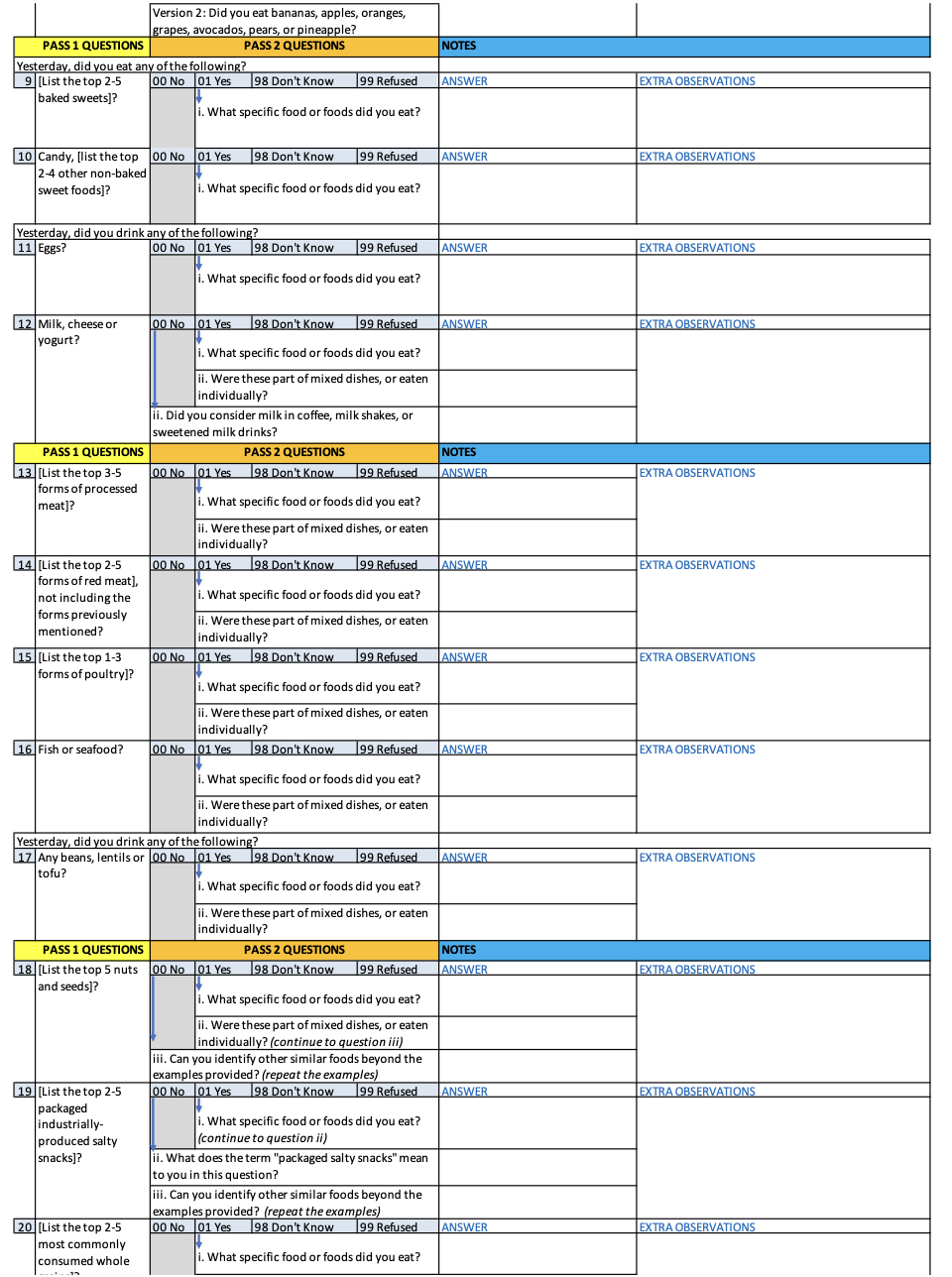


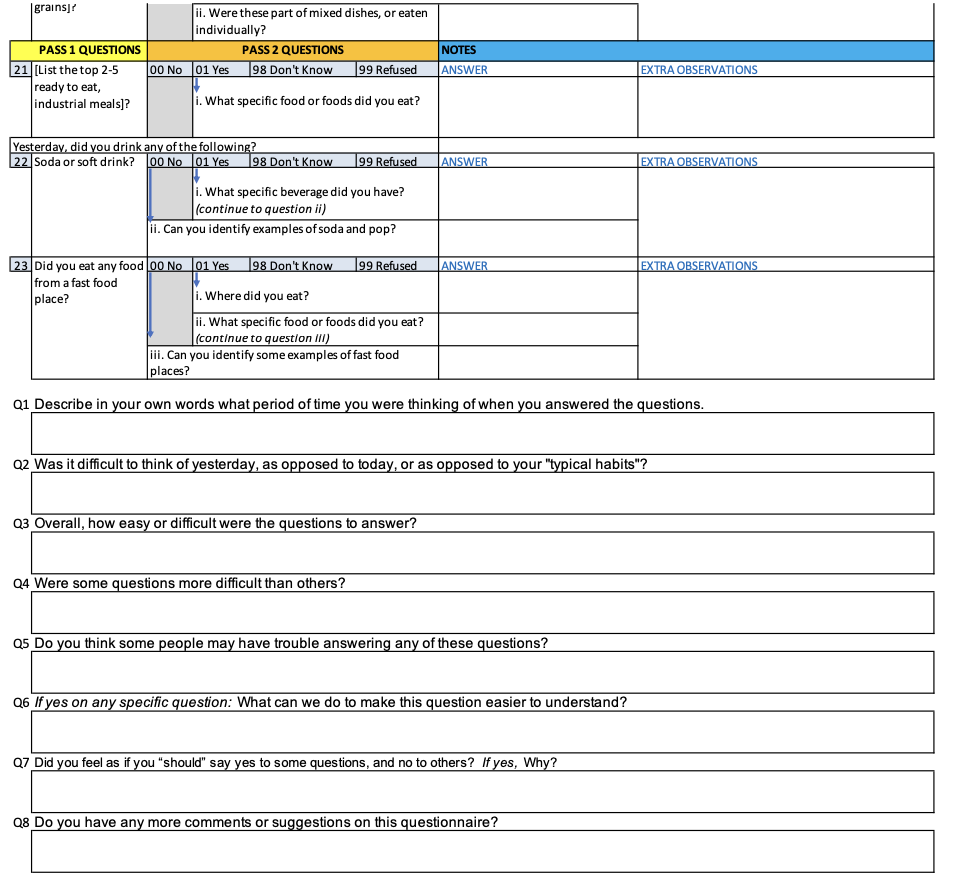


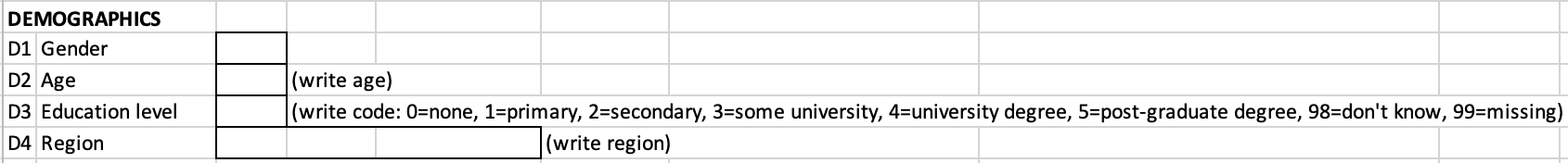


## Cognitive Testing Questionnaire A implemented in greater São Paulo, Brazil


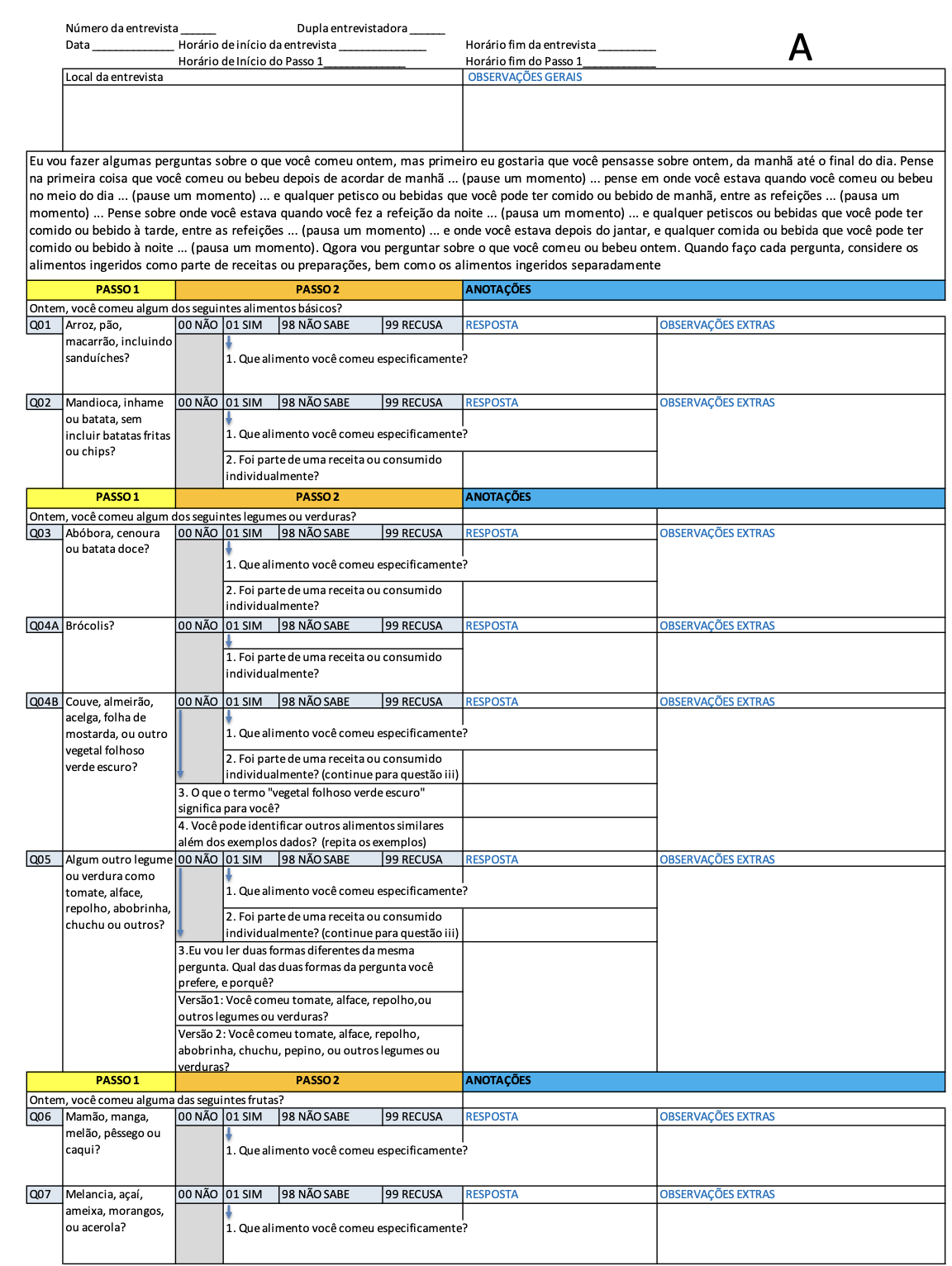


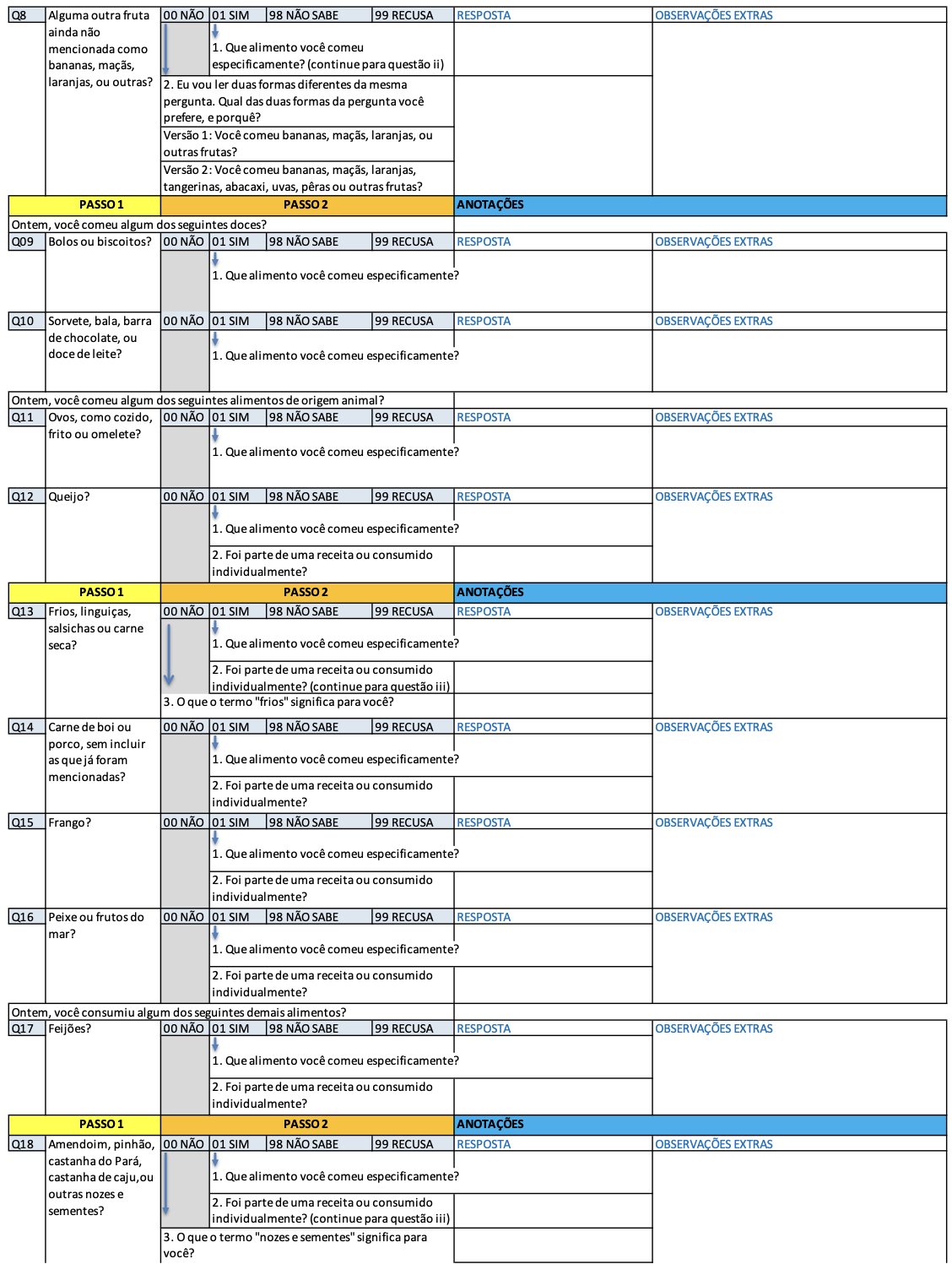


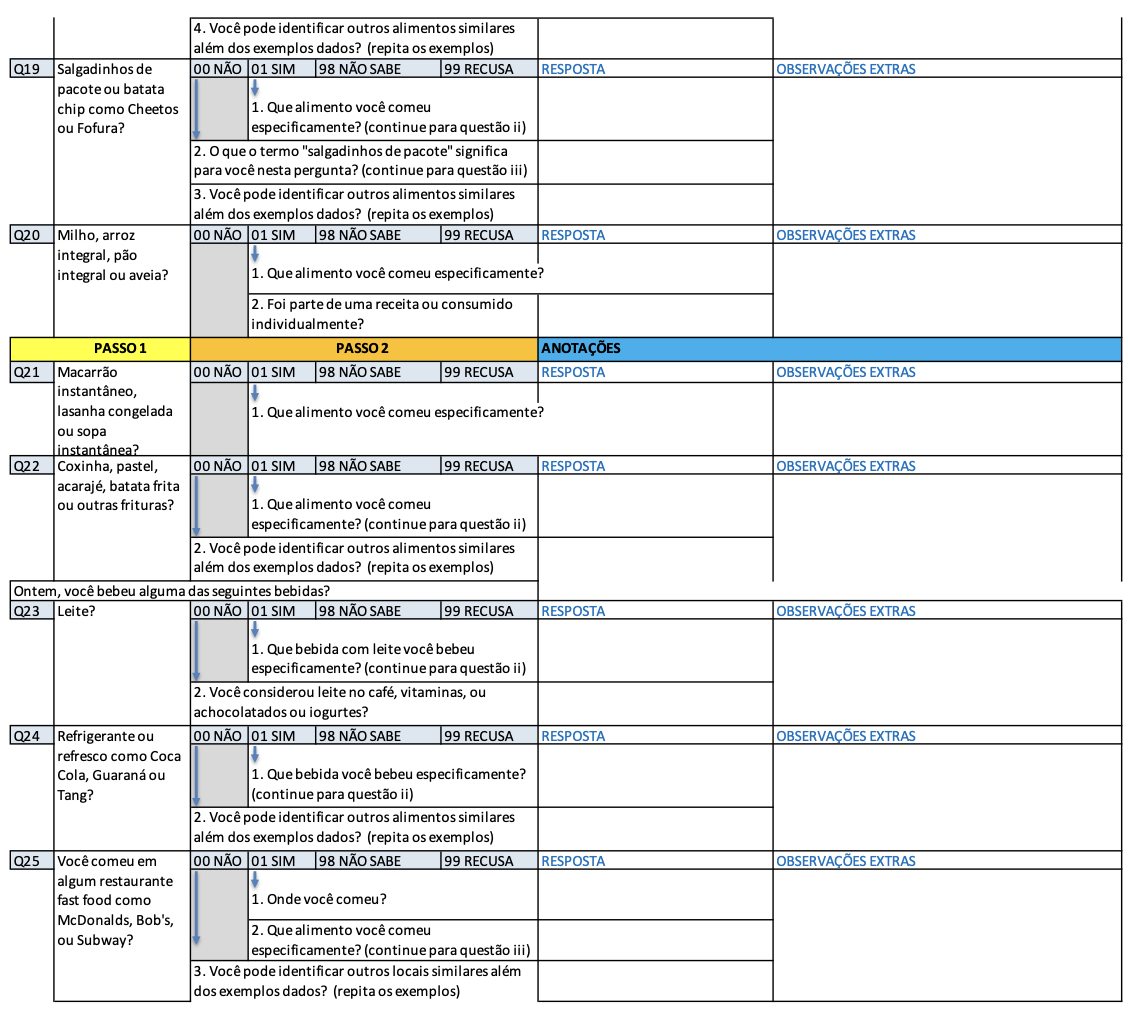


## Cognitive Testing Questionnaire B implemented in greater São Paulo, Brazil


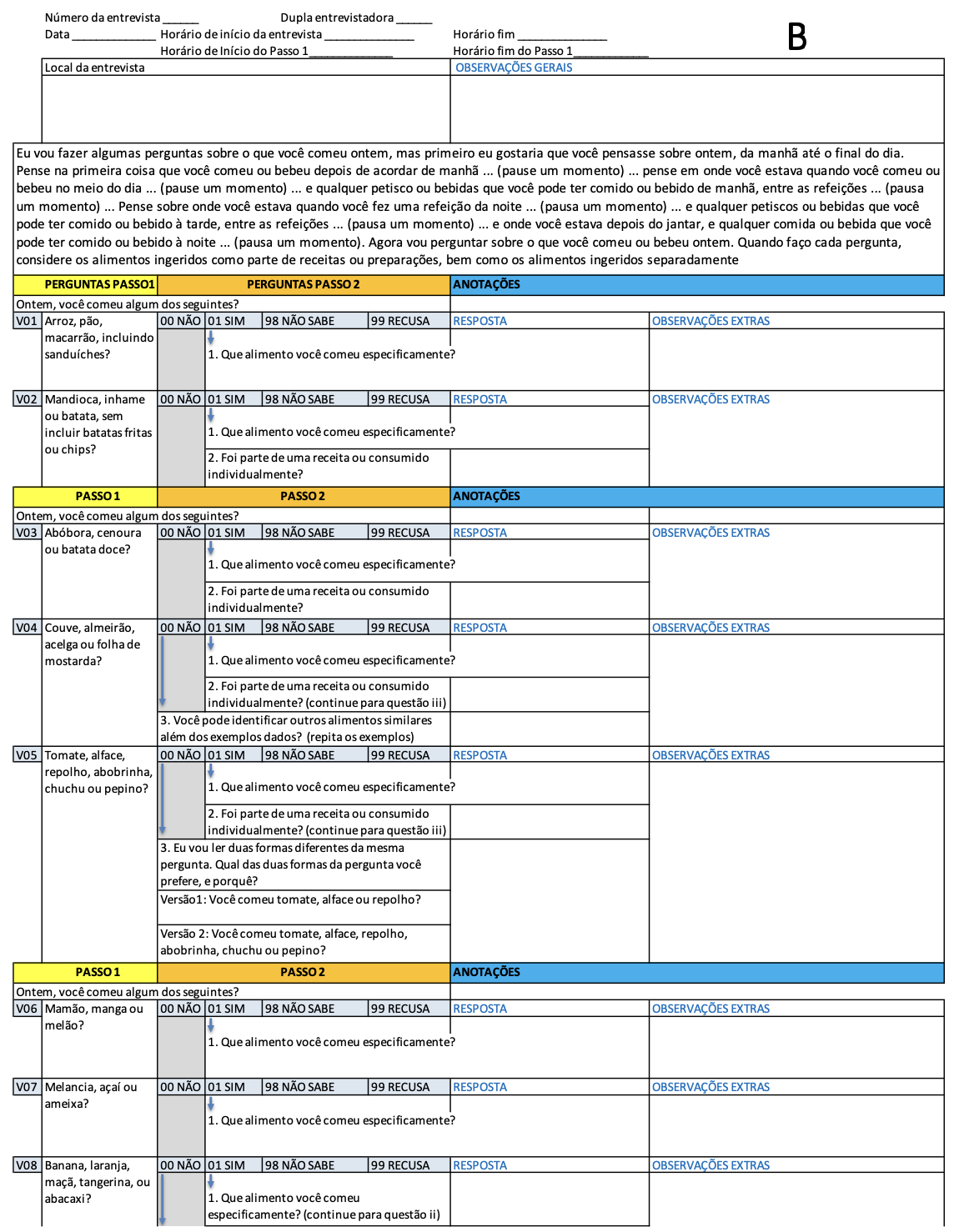


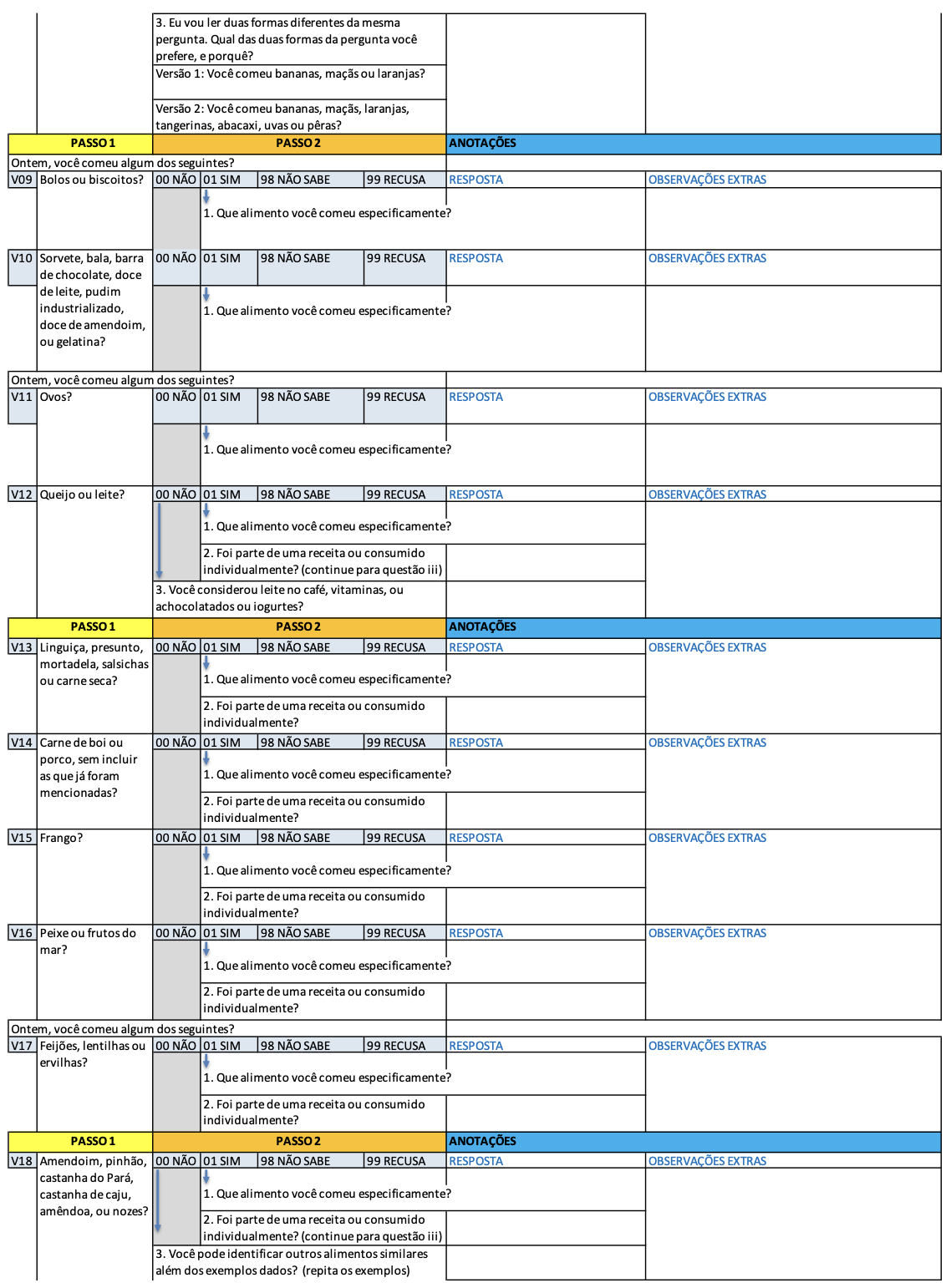


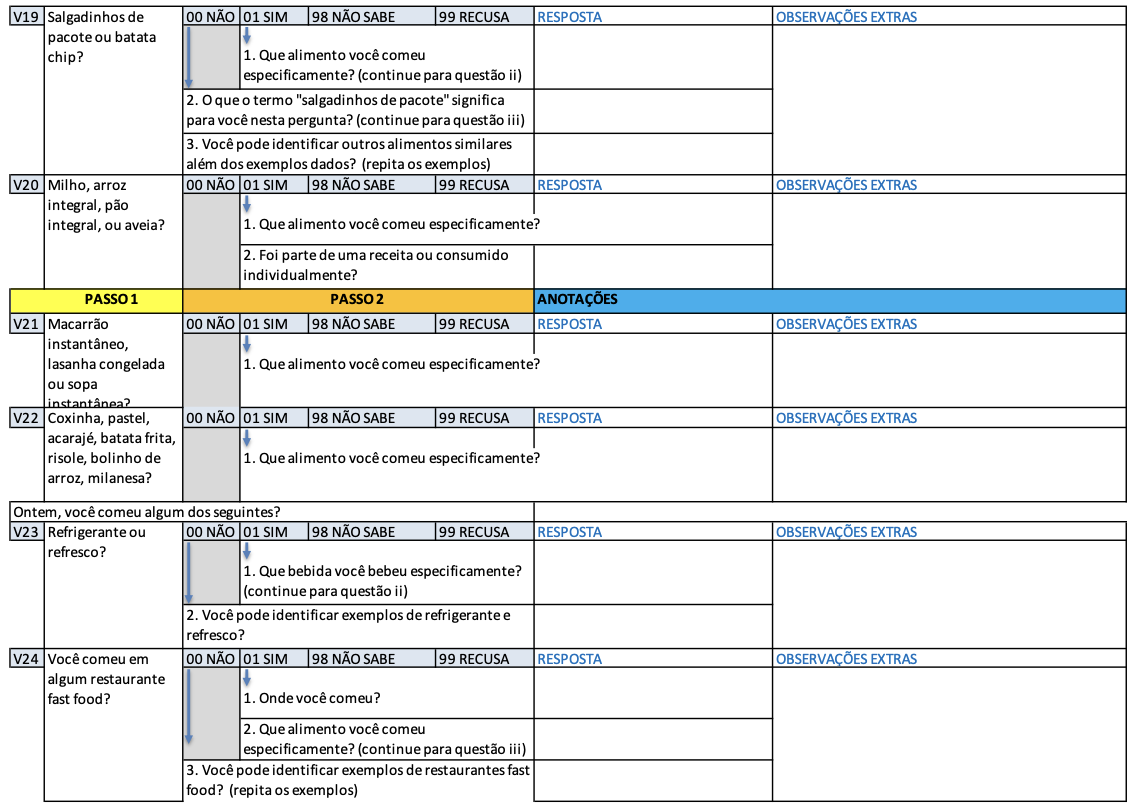


**Versão A & B**

Questões para perguntar depois do PASSO 2:

1. Descreva em suas próprias palavras o período de tempo no qual você estava pensando enquanto respondia as perguntas.
2. Foi difícil pensar no dia de ontem, em oposição ao dia de hoje, ou em oposição nos seus hábitos típicos?
3. De modo geral, o quão difícil foi de responder as perguntas?
4. Algumas perguntas foram mais difíceis que outras?
5. Você acha que algumas pessoas podem achar difícil responder a estas perguntas?
6. Se sim em alguma das perguntas especificamente, o que nós podemos fazer para torná-las mais fácil de responder?
7. Você sentiu, de algum forma, que tinha que responder SIM para algumas perguntas, e NÃO a outras? Se sim, porquê?
8. Você tem mais algum comentário ou sugestão para melhorar este questionário?

Perguntas a serem respondidas ao final da entrevista:

1. Gênero: (Circule apenas, NÃO pergunte)

| **Masculino** | **Feminino** |
| --- | --- |
| 1 | 2 |

1. Respondente mora em zona: (Circule apenas, NÃO pergunte)

| **Urbana** | **Rural** |
| --- | --- |
| 1 | 2 |

1. Por favor me fale sua idade.

| **Escreva em anos:** |  |
| --- | --- |
| (Refused) | 0 |

1. Até que ano da escola você estudou? *(Entrevistador: o respondente deve ter concluído até o último ano do curso para ser respondido que o alcançou)*

|  | **circule uma resposta** |
| --- | --- |
| Sem educação formal | 0 |
| Primário / Fundamental I | 1 |
| Ginasial / Fundamental II | 2 |
| Colegial /Ensino Médio | 3 |
| Superior completo | 4 |
| Pós graduação (mestrado/doutorado) | 5 |
| (Não sabe) | 98 |
| (Recusa) | 99 |

1. Você é de que região do país?

|  | **circule uma resposta** |
| --- | --- |
| Norte | 0 |
| Nordeste | 1 |
| Centro-oeste | 2 |
| Sudeste | 3 |
| Sul | 4 |
| Outra | 5 |
| (Não sabe) | 98 |
| (Recusa) | 99 |

1. Quantos salários mínimos é sua renda MENSAL individual, antes dos impostos? Lembre que um salário mínimo no Brasil atualmente é R$954,00. Por favor inclua renda em salários, recebimentos da família, produção agrícola, e qualque outra fonte.. Novamente, por favor me fale sua renda total MENSAL individual, em salários mínimos:

|  | **circule uma resposta** |
| --- | --- |
| (sem renda) | 0 |
| Menos que um salário mínimo | 1 |
| Um salário mínimo | 2 |
| Dois salários mínimos | 3 |
| Mais do que dois salários mínimos | 4 |
| (Não sabe a renda) | 98 |
| (Se recusa a responder) | 99 |

## Region of origin of cognitive interview respondents

| **Brazil** | | |
| --- | --- | --- |
| North/Northeast | 16 | 33% |
| South/Southeast/Midwest | 32 | 66% |
| **Egypt (residing in NYC)** | | |
| Alexandria | 2 | 33% |
| Al-Saeed | 1 | 17% |
| Delta | 1 | 17% |
| **Iran (residing in NYC)** | | |
| Tehran | 3 | 50% |
| Shiraz | 1 | 17% |
| Hamadan | 2 | 33% |
| **China (residing in NYC)** | | |
| Canton | 1 | 8% |
| Zhejiang | 1 | 8% |
| Shanghai | 1 | 8% |
| Guangdong | 1 | 8% |
| Beijing | 1 | 8% |
| Sichuan | 2 | 15% |
| Taiwan | 3 | 23% |
| Liao Ning | 2 | 15% |
| **United States** | | |
| Northeast* | 12 | 100% |

** One US respondent declined to report her region of origin.*

## Cognitive testing responses to qualitative questions

Follow-up questions

*Q.1: Was it difficult to think of yesterday?*

*Q.2: Overall, how easy or difficult were the questions to answer?*

*Q.3: Were some questions more difficult than others?*

*Q.4: Do you think some people may have trouble answering any of these questions?*

*Q.5: Did you feel as if you “should” say yes to some questions, and no to others?*

|  | **Q.1** | | **Q.2** | | **Q.3** | | **Q.4** | | **Q.5** | |
| --- | --- | --- | --- | --- | --- | --- | --- | --- | --- | --- |
|  | **Yes** | **No** | **Easy** | **Diff.** | **Yes** | **No** | **Yes** | **No** | **Yes** | **No** |
| **Brazil** | 2 | 41 | 41 | 2 | 5 | 38 | 16 | 27 | 0 | 43 |
| **China** | 2 | 11 | 12 | 1 | 4 | 9 | 7 | 5 | 0 | 13 |
| **Iran** | 3 | 3 | 6 | 0 | 0 | 5 | 2 | 3 | 0 | 6 |
| **USA** | 4 | 8 | 10 | 2 | 3 | 8 | 3 | 9 | 4 | 9 |
| **Egypt** | 1 | 5 | 6 | 0 | 0 | 6 | 0 | 6 | 0 | 6 |
| missing | 3 | | 3 | | 5 | | 5 | | 3 | |
| **TOTAL** | **12** | **68** | **75** | **5** | **12** | **66** | **28** | **50** | **4** | **77** |
| **Percentage** | **15%** | **85%** | **94%** | **6%** | **15%** | **85%** | **36%** | **64%** | **5%** | **95%** |

## Quantitative pilot test in the Gallup World Poll: Differences between questionnaire versions A and B implemented in a national sample in Brazil, 2018

|  | | | | |
| --- | --- | --- | --- | --- |
| Training | Speak slowly for questions that have a lot of examples. | | | |
| Question | Version A  All Closed-list questions. | | Version B  SHORTER (3 questions fewer),  4 OPEN-LIST QUESTIONS | |
| Introduction | I will ask you some questions about the food you ate yesterday, and your answers should be yes or no. First I would like you just to think about yesterday, from the morning until the end of the day. Think to yourself about the first thing you ate or drank after you woke up in the morning …(pause a moment)…think about where you were when you had any food or drink in the middle of the day …(pause a moment)…and any snacks or drinks you may have had in the morning, between meals …(pause a moment)…Think about where you were when you had an evening meal …(pause a moment)…and any snacks or drinks you may have had in the afternoon, between meals …(pause a moment)…and where you were after dinner, and any food or drink you may have had in the evening...(pause a moment). Now I will ask you about what you had to eat or drink yesterday. Please listen to the list foods I read in each question, and if you ate *any one of them*, say yes: For example if I ask ‘Did you eat rice, pasta, or bread’ and you only ate rice, you would answer ‘yes.’ When I ask you each question, please consider foods eaten in mixed dishes, where these food were included as an ingredient, mixed with other foods, as well as foods eaten on their own, and please respond “yes” or “no.” | | | |
| STAPLE FOODS | | | | |
| 1. | Rice, pasta, or bread, including sandwiches? | | Exclude the question | |
| 2. | Potato, cassava, or yam? | | Exclude the question | |
| 3. | Beans or lentils? | | | |
| VEGETABLES | | | | |
| 4. | Pumpkin, carrot, or sweet potato? | | | |
| 5. | CLOSED  Kale, broccoli, almeirao, chard, or mustard greens? | | OPEN  Broccoli, kale, almeirao, chard, mustard greens, or other dark green leaves? | |
| 6. | CLOSED  Lettuce, tomato, cabbage, beet, zucchini, chuchu, or cucumber? | | OPEN  “Other vegetables not already mentioned, such as lettuce, tomato, cabbage, beet, zucchini, chuchu, cucumber, or others?” | |
| FRUITS | | | | |
| 7. | Papaya, mango, peach, or persimmon? | | | |
| 8. | Watermelon, acai, plum, strawberry, or acerola? | | | |
| 9. | Orange or tangerine? | | (incorporated into question 10) | |
| 10. | CLOSED  Banana, apple, pineapple, grape, or pear? | | OPEN  “Other fruits not already mentioned, such as banana, orange, tangerine, apple, pineapple, grape, pear, or others?” | |
| SWEETS | | | | |
| 11. | Cakes, cookies, sweet tortas, or sweet breads? | | | |
| 12. | Ice cream, candy, chocolate bars, doce de leite, pudding, peanut sweets, or gelatin? | | | |
| FOODS FROM ANIMAL ORIGEN | | | | |
| 13. | Eggs? | | | Eggs, such as boiled, fried, or in an omelet? |
| 14. | Cheese? | | | |
| 15. | Sausages, ham, mortadela (bologna), hot dogs or dried meats? | | | |
| 16. | Beef or pork? | | | |
| 17. | Chicken? | | | |
| 18. | Fish or seafood? | | | |
| OTHER FOODS | | | | |
| 19. | Peanuts, pine nuts, Brazil nuts, cashews, almonds, or walnuts? | | | |
| 20. | Packaged salty snacks such as potato chips, Cheetos or Fofura? | | | |
| 21. | Corn, brown rice, wholegrain bread, or oats? | | | |
| 22. | Instant noodles, frozen lasagna, or instant soup? | | | |
| 23. | CLOSED  Pastel, coxinha, enroladinho, quibe, french fries, fried cassava, or milanesa? | OPEN  Pastel, coxinha, enroladinho, quibe, french fries, fried cassava, milanesa, or other deep fried foods? | | |
| BEVERAGES | | | | |
| 24. | Milk? | | | |
| 25. | Soda or soft drinks, such as Coca Cola, Guarana, or Tang? | | | |
|  | | | | |
| 26. | Did you eat in any place like McDonald’s, Bob’s, Subway, Habib’s, or Burger King? | | | |

## Quantitative pilot test in the Gallup World Poll: Questionnaire version A and B as implemented in the Gallup World Poll in Brazil, 2018

| **QUESTIONÁRIO A**  Eu vou te fazer algumas perguntas sobre os alimentos que você comeu ontem, e as suas respostas devem ser SIM ou NÃO. Primeiro eu gostaria que você pensasse sobre ontem, da manhã até o final do dia. Pense para si mesmo sobre a primeira coisa que você comeu ou bebeu depois de acordar de manhã ... (pausar um momento) ... pense em onde você estava quando comeu ou bebeu alguma coisa no meio do dia ... (pare um pouco) ... e qualquer lanchinho ou bebida que você possa ter consumido de manhã, entre as refeições… (pause um momento)… Pense sobre onde você estava quando você fez a refeição da noite… (pause um momento)… e qualquer lanchinho ou bebida que você possa ter consumido a tarde, entre as refeições ... (pausa um momento) ... e onde você estava depois do jantar, e qualquer comida ou bebida que você pode ter consumido à noite ... (pausa um momento).  Agora vou perguntar sobre o que você comeu ou beber ontem. Por favor, ouça a lista de alimentos que eu leio em cada pergunta, e se você comeu qualquer um deles, diga sim: Por exemplo, se eu perguntasse 'você comeu arroz, massa ou pão' e você só comeu arroz, você responderia ‘Sim’. Quando eu fizer cada pergunta, por favor, considere os alimentos ingeridos em preparações com estes alimentos como ingredientes de receitas, misturados com outros alimentos, assim como os alimentos consumidos separados, e responda' sim 'ou' não '. |
| --- |
| \| Ontem, você comeu algum dos seguintes alimentos básicos? \| \| \| --- \| --- \| \| AQ01 \| Arroz, macarrão ou pão, incluindo sanduíches? \| \| AQ02 \| Batata, mandioca ou inhame? \| \| AQ03 \| Feijão ou lentilha? \| \| Ontem, você comeu algum dos seguintes legumes ou verduras? \| \| \| AQ04 \| Abóbora, cenoura ou batata doce? \| \| AQ05 \| Brócolis, couve, almeirão, acelga ou folha de mostarda? \| \| AQ06 \| Alface, tomate, repolho, beterraba, abobrinha, chuchu ou pepino? \| \| Ontem, você comeu alguma das seguintes frutas? \| \| \| AQ07 \| Mamão, manga, pêssego ou caqui? \| \| AQ08 \| Melancia, açaí, ameixa, morango, ou acerola? \| \| AQ09 \| Laranja ou tangerina? \| \| AQ10 \| Banana, maçã, abacaxi, uva ou pêra? \| \| Ontem, você comeu algum dos seguintes alimentos doces? \| \| \| AQ11 \| Bolo, biscoito doce, torta doce ou pão doce? \| \| AQ12 \| Sorvete, bala, barra de chocolate, doce de leite, pudim industrializado, doce de amendoim ou gelatina? \| \| Ontem, você comeu algum dos seguintes alimentos de origem animal? \| \| \| AQ13 \| Ovo? \| \| AQ14 \| Queijo? \| \| AQ15 \| Linguiça, presunto, mortadela, salsicha ou carne seca? \| \| AQ16 \| Carne de boi ou porco? \| \| AQ17 \| Frango? \| \| AQ18 \| Peixe ou frutos do mar? \| \| Ontem, você consumiu algum dos seguintes alimentos? \| \| \| AQ19 \| Amendoim, pinhão, castanha do Pará, castanha de caju, amêndoas ou nozes? \| \| AQ20 \| Salgadinhos de pacote como batata chips, Cheetos ou Fofura? \| \| AQ21 \| Milho, arroz integral, pão integral ou aveia? \| \| AQ22 \| Macarrão instantâneo, lasanha congelada ou sopa instantânea? \| \| AQ23 \| Pastel, coxinha, enroladinho, quibe, batata frita, mandioca frita, ou milanesa? \| \| Ontem, você bebeu alguma das seguintes bebidas? \| \| \| AQ24 \| Leite? \| \| AQ25 \| Refrigerante ou refresco como Coca Cola, Guaraná ou Tang? \| \|  \| \| \| AQ26 \| Você comeu em algum lugar como McDonald’s, Bob's, Burger King, Habib’s ou Subway? \| |
|  |
| **QUESTIONÁRIO B**  Eu vou te fazer algumas perguntas sobre os alimentos que você comeu ontem, e as suas respostas devem ser SIM ou NÃO. Primeiro eu gostaria que você pensasse sobre ontem, da manhã até o final do dia. Pense para si mesmo sobre a primeira coisa que você comeu ou bebeu depois de acordar de manhã ... (pausar um momento) ... pense em onde você estava quando comeu ou bebeu alguma coisa no meio do dia ... (pare um pouco) ... e qualquer lanchinho ou bebida que você possa ter consumido de manhã, entre as refeições… (pause um momento)… Pense sobre onde você estava quando você fez a refeição noturna… (pause um momento)… e qualquer lanchinho ou bebida que você possa ter consumido a tarde, entre as refeições ... (pausa um momento) ... e onde você estava depois do jantar, e qualquer comida ou bebida que você pode ter consumido à noite ... (pausa um momento).  Agora vou perguntar sobre o que você comeu ou beber ontem. Por favor, ouça a lista de alimentos que eu leio em cada pergunta, e se você comeu qualquer um deles, diga sim: Por exemplo, se eu perguntasse 'você comeu arroz, massa ou pão' e você só comeu arroz, você responderia ‘Sim’. Quando eu fizer cada pergunta, por favor, considere os alimentos ingeridos em pratos mistos, assim como os alimentos consumidos separados, e responda' sim 'ou' não '. |
| \| Ontem você comeu: \| \| \| --- \| --- \| \| BQ01 \| Feijão ou lentilha? \| \| Ontem, você comeu algum dos seguintes legumes ou verduras? \| \| \| BQ02 \| Abóbora, cenoura ou batata doce? \| \| BQ03 \| Brócolis, couve, almeirão, acelga, folha de mostarda, ou outra folha verde escura? \| \| BQ04 \| Algum outro legume ou verdura ainda não mencionado como alface, tomate, repolho, beterraba, abobrinha, chuchu, pepino ou outros? \| \| Ontem, você comeu alguma das seguintes frutas? \| \| \| BQ05 \| Mamão, manga, pêssego ou caqui? \| \| BQ06 \| Melancia, açaí, ameixa, morangos, ou acerola? \| \| BQ07 \| Alguma outra fruta ainda não mencionada como banana, laranja, tangerina, maçã, abacaxi, uva, pêra ou outras? \| \| Ontem, você comeu algum dos seguintes doces? \| \| \| BQ08 \| Bolo, biscoito doce, torta doce ou pão doce? \| \| BQ09 \| Sorvete, bala, barra de chocolate, doce de leite, pudim industrializado, doce de amendoim ou gelatina? \| \| Ontem, você comeu algum dos seguintes alimentos de origem animal? \| \| \| BQ10 \| Ovos, como cozido, frito ou omelete? \| \| BQ11 \| Queijo? \| \| BQ12 \| Linguiça, presunto, mortadela, salsicha ou carne seca? \| \| BQ13 \| Carne de boi ou porco? \| \| BQ14 \| Frango? \| \| BQ15 \| Peixe ou frutos do mar? \| \| Ontem, você consumiu algum dos seguintes alimentos? \| \| \| BQ16 \| Amendoim, pinhão, castanha do Pará, castanha de caju, amêndoas ou nozes? \| \| BQ17 \| Salgadinhos de pacote como batata chip, Cheetos ou Fofura? \| \| BQ18 \| Milho, arroz integral, pão integral ou aveia? \| \| BQ19 \| Macarrão instantâneo, lasanha congelada ou sopa instantânea? \| \| BQ20 \| Pastel, coxinha, enroladinho, quibe, batata frita, mandioca frita, milanesa, ou outras frituras? \| \| Ontem, você bebeu alguma das seguintes bebidas? \| \| \| BQ21 \| Leite? \| \| BQ22 \| Refrigerante ou refresco como Coca Cola, Guaraná ou Tang? \| \|  \| \| \| BQ23 \| Você comeu em algum lugar como McDonald’s, Bob's, Burger King, Habib’s ou Subway? \| |

## Supplementary Table 1. Sample characteristics of the two questionnaire versions implemented in the Gallup pilot test in Brazil, 2018

|  | **Form A (N=519)** | | | **Form B (N=481)** | | |
| --- | --- | --- | --- | --- | --- | --- |
|  | **Sample % Unweighted** | **Target %** | **Sample % Weighted** | **Sample % Unweighted** | **Target %** | **Sample % Weighted** |
| **Age** | | | | | | |
| 15-29 | 24.08 | 35.16 | 34.36 | 23.08 | 35.16 | 33.17 |
| 30-44 | 29.67 | 28.67 | 27.95 | 26.61 | 28.67 | 27.67 |
| 45-59 | 23.70 | 21.28 | 21.60 | 24.74 | 21.28 | 21.89 |
| 60+ | 22.54 | 14.89 | 16.09 | 25.57 | 14.89 | 17.28 |
| **Gender** | | | | | | |
| Male | 42.39 | 47.80 | 45.95 | 45.95 | 47.80 | 46.68 |
| Female | 57.61 | 52.20 | 54.05 | 54.05 | 52.20 | 53.32 |
| **Education** | | | | | | |
| No formal education/  Primary Incomplete | 29.09 | 46.10 | 42.85 | 26.20 | 46.10 | 41.17 |
| Primary Complete/  High School Incomplete | 31.41 | 18.80 | 20.80 | 33.47 | 18.80 | 21.34 |
| High School Complete/ Higher Education | 39.50 | 35.10 | 36.35 | 40.33 | 35.10 | 37.48 |
| **Region** | | | | | | |
| South | 11.37 | 14.80 | 14.50 | 18.92 | 14.80 | 15.73 |
| Southeast | 42.00 | 43.60 | 43.38 | 39.92 | 43.60 | 43.98 |
| Centre-West | 7.71 | 7.20 | 6.66 | 8.32 | 7.20 | 7.68 |
| North | 8.86 | 7.30 | 7.40 | 7.07 | 7.30 | 7.45 |
| Northeast | 30.06 | 27.10 | 28.06 | 25.78 | 27.10 | 25.16 |
| **Urbanicity** | | | | | | |
| Urban | 83.82 | 84.00 | 83.69 | 88.36 | 84.00 | 85.25 |
| Rural | 16.19 | 16.00 | 16.31 | 11.64 | 16.00 | 14.75 |

## Supplementary Table 2. Prevalence levels and confidence intervals of the two questionnaire versions implemented in the Gallup pilot test in Brazil, 2018

|  | Food group | Version A (closed-ended) | Version B (open-ended) | Difference in prevalence (Form A minus Form B) (percentage points) | p-value* |
| --- | --- | --- | --- | --- | --- |
| 1 | Foods made from grains | 94% | - |  | - |
| 2 | Starchy white roots, tubers, and plantains | 38% | - |  | - |
| 3 | Legumes | 84% | 85% | -0.1 | 0.98 |
| 4 | Vitamin A rich orange vegetables | 39% | 37% | 2.8 | 0.47 |
| 5 | Dark green leafy vegetables** | **18%** | **27%** | -9.1 | 0.04 |
| 6 | Other vegetables** | **57%** | **63%** | -6.6 | 0.10 |
| 7 | Vitamin A-rich fruits | 20% | 17% | 3.2 | 0.28 |
| 8 | Red, purple, or blue fruits | 18% | 13% | 4.7 | 0.08 |
| 9-10 | Other fruits (including citrus)** | **64%** | **63%** | 0.6 | 0.96 |
| 11 | Baked or grain-based sweets | 47% | 55% | -7.4 | 0.11 |
| 12 | Other sweets | 33% | 33% | 0.2 | 0.94 |
| 13 | Eggs | 44% | 44% | 0.4 | 0.79 |
| 14 | Cheese | 29% | 30% | -1.1 | 0.76 |
| 15 | Processed meats | 41% | 43% | -2.2 | 0.71 |
| 16 | Red meat | 55% | 58% | -3.0 | 0.46 |
| 17 | Poultry | 47% | 47% | -0.2 | 0.95 |
| 18 | Fish and seafood | 13% | 7% | 6.2 | 0.005 |
| 19 | Nuts and seeds | 10% | 9% | 0.7 | 0.71 |
| 20 | Packaged ultra-processed salty snacks | 13% | 15% | -1.9 | 0.48 |
| 21 | Whole grains | 35% | 32% | 3.5 | 0.31 |
| 22 | Instant noodles and other ready meals | 11% | 16% | -5.0 | 0.02 |
| 23 | Deep fried foods** | **20%** | **21%** | -1.3 | 0.62 |
| 24 | Milk | 56% | 50% | 6.2 | 0.17 |
| 25 | Soft drinks (sodas) | 44% | 46% | -1.5 | 0.61 |
| 26 | Fast food | 4% | 4% | 0.5 | 0.52 |

* p-values of <0.002 are assigned significance, after Bonferroni adjustment for multiple comparisons (0.05/23). By this criterion, no difference between A and B was significant.

**questions which were closed-ended in Version A, and open-ended in Version B.

## Supplementary Figure 1. Proportion responding “yes” to each question in Form A and B, in increasing order of prevalence


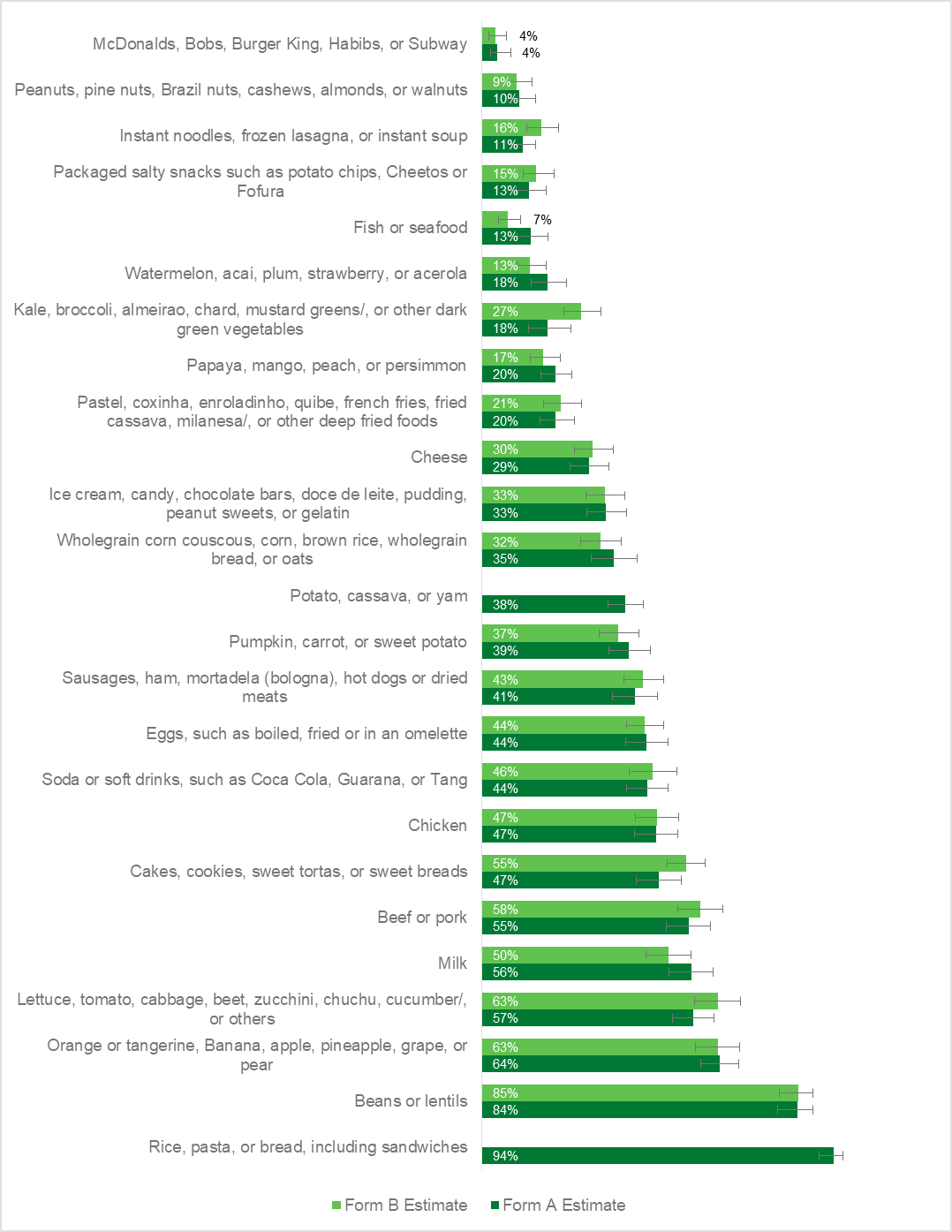

Supplement: Multimedia component 1 [file mmc1.docx]
